# Ambient gold-catalyzed *O*-vinylation of cyclic 1,3-diketone: A vinyl ether synthesis

**DOI:** 10.3762/bjoc.9.288

**Published:** 2013-11-18

**Authors:** Yumeng Xi, Boliang Dong, Xiaodong Shi

**Affiliations:** 1 C. Eugene Bennett Department of Chemistry, West Virginia University, Morgantown, West Virginia 26506, United States

**Keywords:** alkyne, copper salt, diketone, gold catalysis, vinyl ether

## Abstract

Gold-catalyzed O-vinylation of cyclic 1,3-diketones has been achieved for the first time, which provides direct access to various vinyl ethers. A catalytic amount of copper triflate was identified as the significant additive in promoting this transformation. Both aromatic and aliphatic alkynes are suitable substrates with good to excellent yields.

## Introduction

The past decade has witnessed the fast growth of homogeneous gold catalysis as one of the important branches in transition-metal chemistry [1–9]. The utility of cationic gold(I) complexes as π-carbophilic acids toward alkyne and allene activation renders them as essential tools in organic synthesis. Generally with the unique reactivity and mild reaction conditions, this type of transformation has found widespread applications in complex molecule synthesis [10–11]. Among those reactions, the gold-catalyzed hydration of alkynes is regarded as one of the signature reactions in the field (known as Teles hydration) [12]. This reaction usually utilized the combination of methanol and water, wherein methanol served as the nucleophile to attack the triple bond, forming the vinyl ether intermediate. This vinyl ether then collapsed to give a ketone as the final product [13–17].

A vinyl ether is a common and versatile building block in organic synthesis as well as polymer chemistry. Typical methods for the preparation of a vinyl ether involve elimination, olefination of esters, addition of alcohols to alkynes, as well as transition metal-mediated cross-coupling reactions [18]. Based on the π-carbophilicity of gold(I), the addition of alcohol to alkyne should provide direct access to the corresponding vinyl ether. However, most of the reported gold-catalyzed O-nucleophile additions to alkynes are intramolecular reactions. No general protocol for vinyl ether synthesis using gold has been reported to date [17]. Nevertheless, there have been examples regarding the intermolecular addition of carboxylic acids [19–20], phenols [21–22] as well as phosphoric acids [23–25] to alkynes. In this context, we report a gold(I)-catalyzed O-vinylation of 1,3-diketones with unactivated alkynes at ambient temperature.

## Results and Discussion

**Scheme 1:**
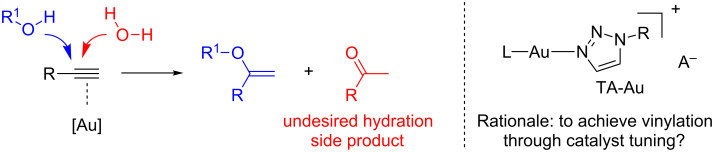
Intermolecular O-addition to alkynes: challenge and opportunity.

As indicated in Scheme 1, the main challenge for the intermolecular O-nucleophile addition to alkynes is the competitive hydration side reaction. Although, theoretically, strictly anhydrous conditions shall prevent the water addition, new effective catalytic systems that can avoid the “precautionary” treatment of solvents and substrates are much more practical and highly desirable.

From literature reported examples, a gold activated water intermediate, [L-Au-(H_2_O)]^+^, has been proposed in helping the water addition to the alkyne besides the typical π-acid activation [12–17]. Our group has developed the 1,2,3-triazole coordinated gold complexes (TA-Au) as stable catalysts for alkyne activation in the past several years [26–30]. Considering that TA-Au complexes might have different binding ability toward water activation, we wondered whether the intermolecular O-addition could be achieved through ligand tuning. In this report, we focus on the cyclic 1,3-diketone nucleophiles due to A) the transformation is challenging and has never been reported in the past, B) vinyl ether products are highly functional materials, which can be easily extended to complex molecules through simple transformations.

1,3-Cyclohexanedione and phenylacetylene were used as the model substrates for the evaluation of various gold catalysts. As shown in Table 1, the use of Ph_3_PAuCl/AgOTf (5%) gave an acceptable yield (59%) of the desired vinyl addition product **3a**. Thermally-stable Ph_3_PAu(TA)OTf (TA-Au, 5%) slightly improved the performance, yielding 63% of the desired product and less hydration byproduct **4**. When changing the primary ligand from triphenylphosphine to a NHC, the yield slightly decreased. However, the corresponding TA-Au complexes indicated significantly improved selectivity towards the diketone addition over the hydration (Table 1, entry 4). Finally, the application of the XPhos ligand and the corresponding TA-Au complex largely promoted this reaction, giving **3a** in 85% yield with 12% hydration byproduct. Notably, the reaction occurred at room temperature with no need for “careful” condition control (open to the air and untreated solvent). The fact that TA-Au complexes generally gave improved selectivity of diketone addition over hydration (compared with the corresponding [L-Au]^+^ complexes) supported our hypothesis and made the TA-Au catalysts important for this transformation. Slightly increasing the ratio of alkyne to 1.2 equivalents (relative to the nucleophile) increased the formation of the desired vinyl ester to a near quantitative yield (98% NMR yield, 95% isolated yield), even with only 1% catalyst loading.

**Table 1:** Screening of gold catalysts.^a,b^

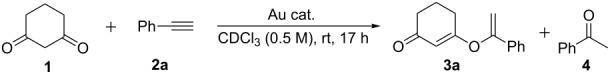							

Entry	[Au] cat.	**2a** (equiv)	Loading	Time (h)	Conversion of **2a** (%)	Yield of **3a** (%)	Yield of **4** (%)

1	PPh_3_AuCl/AgOTf	1.0	5%	17	81	59	17
2	PPh_3_Au(TA)OTf	1.0	5%	17	82	63	11
3	IPrAuCl/AgOTf	1.0	5%	17	100	55	34
4	IPrAu(TA)OTf	1.0	5%	17	64	50	5
5	XPhosAuCl/AgOTf	1.0	5%	10	100	76	22
6	XPhosAu(TA)OTf	1.0	5%	10	100	85	12
7	XPhosAu(TA)OTf	1.2	5%	10	100	99	n.d.
8	XPhosAu(TA)OTf	1.2	3%	17	100	99	n.d.
9	XPhosAu(TA)OTf	1.2	1%	20	100	98 (95)	n.d.

^a^General conditions: **1** (0.2 mmol), **2a** (1 or 1.2 equiv), and catalyst in CDCl_3_ (0.4 mL), rt. ^b^Yield and conversion are determined by using 1,3,5-trimethoxybenzene as the internal standard. Isolated yield is given in parenthesis.

**Scheme 2:**
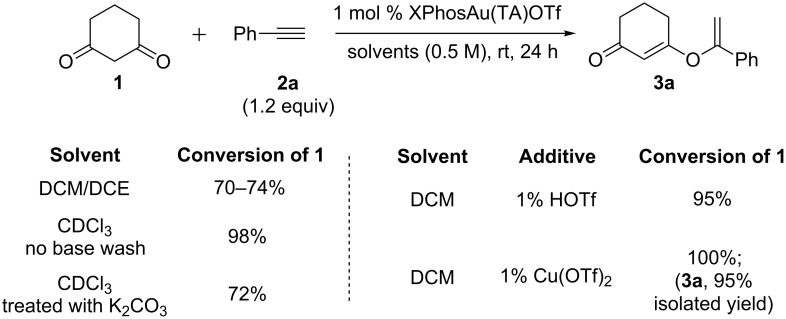
Acid as the critical additive for optimal performance.

Given the encouraging results associated with the XPhosAu-TA catalysts, we explored the reaction scope. However, surprisingly, significantly slower reaction was observed when conducting the reaction in dichloromethane (DCM) or 1,2-dichloroethane (DCE), though selectivity for the diketone addition over hydration was maintained as shown in Table 1. Solvent screening shown in Scheme 2 proved that the trace amount of acid from chloroform decomposition was crucial for the optimal performance, which likely helped the protodeauration. Other Lewis acids have also been screened and the Cu(OTf)_2_ was identified [31] as the optimal choice due to the practical reasons (easy to weigh and less hygroscopic) and excellent reactivity. Cu(OTf)_2_ and HOTf itself could not catalyze the reaction, suggesting that it is indeed a gold-catalyzed process. Finally, it is worth noting that the use of 1 mol % Echavarren catalyst *t-*BuXPhosAu(MeCN)SbF_6_ gave a slightly lower yield (71%), and the combination of 1 mol % *t-*BuXPhosAu(MeCN)SbF_6_ and 1 mol % Cu(OTf)_2_ gave 86% yield (under otherwise identical conditions). This result highlights the synthetic utility of TA-Au catalyst in the transformation.

With this optimal conditions in hand, we embarked on the evaluation of the substrate scope for this transformation. A variety of aromatic alkynes were initially tested as summarized in Table 2.

**Table 2:** Reaction scope of alkynes.^a^

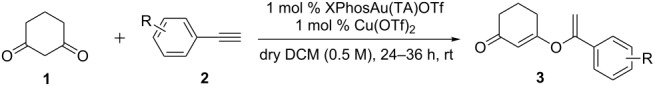					

Entry	Substrate		Product		Yield^b^

1	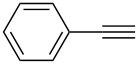	**2a**	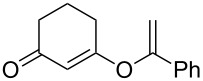	**3a**	95%
2	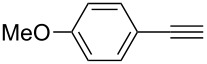	**2b**	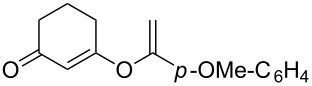	**3b**	88%
3	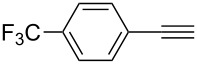	**2c**	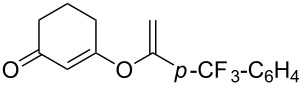	**3c**	89%
4	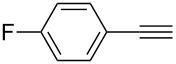	**2d**	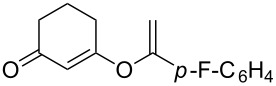	**3d**	87%
5	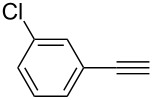	**2e**	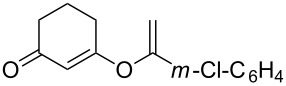	**3e**	59%
6	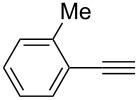	**2f**	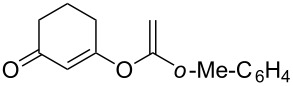	**3f**	68%
7	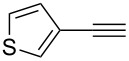	**2g**	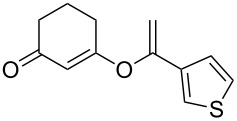	**3g**	86%
8^c^	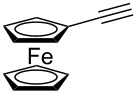	**2h**	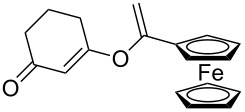	**3h**	70%

^a^General conditions: **1** (0.4 mmol), **2** (1.2 equiv), 1 mol % XPhosAu(TA)OTf and 1 mol % Cu(OTf)_2_ in dry DCM (0.8 mL), rt. ^b^Isolated yield. ^c^**1** (0.4 mmol), **2** (2 equiv), 3 mol % XPhosAu(TA)OTf and 3 mol % Cu(OTf)_2._

This reaction tolerated both electron-rich (**2b**) and electron-deficient (**2c**) alkynes. Aromatic alkynes with substitutents at *meta* (**2e**) and *ortho* (**2f**) positions also worked well, although giving slightly lower yields. Electron-rich heterocycle-containing alkynes (**2g**) were also suitable substrates for this transformation. However, electron-poor alkynes, such as 2- and 3-pyridylacetylenes, did not undergo the reaction, likely caused by the low reactivity of the C–C triple bonds. Interestingly, the addition to the ethynylferrocene gave the corresponding vinyl ether in good yield, which highlighted the mild conditions of this catalytic system and potential applications of this gold catalyst in other metal containing compound syntheses. A series of aliphatic alkynes were also tested for this reaction. Alkynes containing 6-, 5- and 3-membered rings worked well, giving good to excellent yields (Table 3). Notably, the cyclopropylacetylene formed the direct O-addition adduct (**6c**) with no ring opening product observed. In addition, the conjugate enyne could undergo this reaction, giving the interesting electron-rich conjugated diene (**6d**). In some cases, thermodynamically more stable internal alkenes were also observed along with the kinetic product terminal alkene, likely through olefin isomerization [22].

**Table 3:** Reaction scope with aliphatic alkynes.^a^

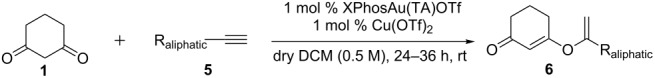					

Entry	Substrate		Product		Yield^b^

1	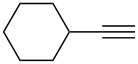	**5a**	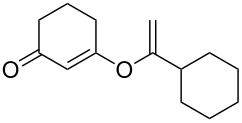	**6a**	89%
2^c^	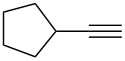	**5b**	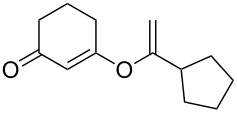	**6b**	80%
3	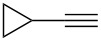	**5c**	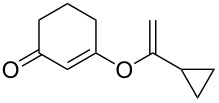	**6c**	86%
4	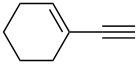	**5d**	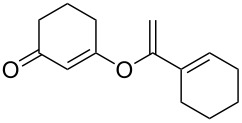	**6d**	86%
5	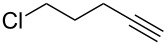	**5e**	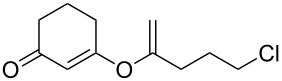	**6e**	67%

^a^General conditions: **1** (0.4 mmol), **5** (2 equiv), 1 mol % XPhosAu(TA)OTf and 1 mol % Cu(OTf)_2_ in dry DCM (0.8 mL), rt. ^b^Isolated yield. ^c^A ratio of 1:0.4 is observed for terminal/internal alkene mixtures. Combined yield.

**Scheme 3:**
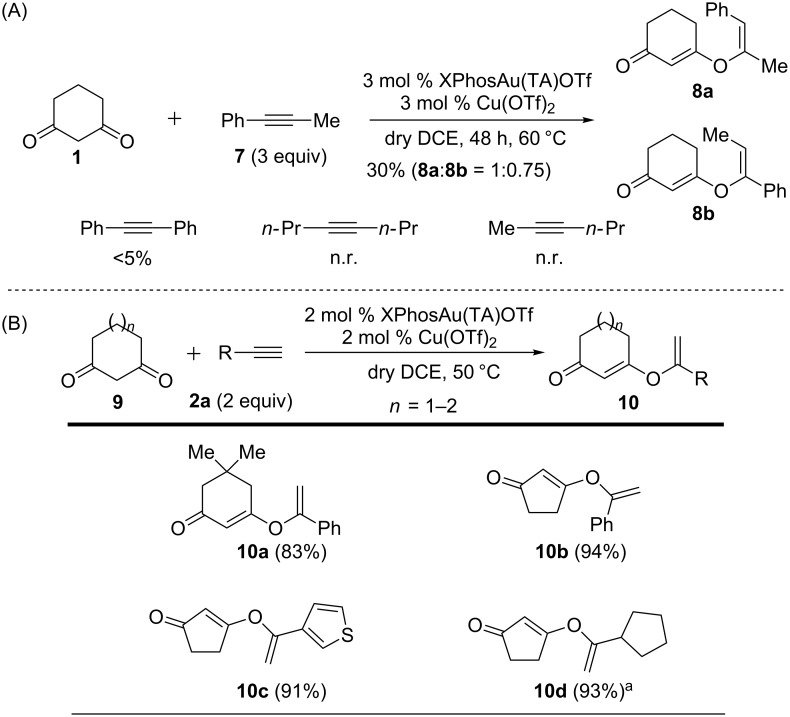
Reactions of internal alkyne and other O-nucleophiles. Isolated yields are given in paranthesis. ^a^A ratio of 1:2.6 is observed for terminal/internal alkene mixtures. Combined yield.

The internal alkyne (1-phenyl-1-propyne), which was usually much less reactive than the terminal alkyne, was also tested. As expected, no reaction occurred at room temperature under the optimal conditions. To our delight, the desired products **8** were obtained while refluxing at 60 °C for 48 h, though in low yield. Two regioisomers were isolated, which were assigned as **8a** and **8b** (Scheme 3A). Diphenylacetylene gave trace amounts of the desired product under the identical conditions.

Several diketones were tested to explore the scope of nucleophiles. The 1,3-cyclohexanedione derivative worked well, giving the vinyl ether **10a** in good yield. The five-membered diketone, 1,3-cyclopentadione could also yield the desired O-vinylation product (**10b–10d**) in excellent yield, under similar conditions. Elevated temperature (50 °C) was required for good results due to the poor solubility of 1,3-cyclopentadione in DCM at room temperature. Finally and notably, all tested acyclic 1,3-diketones as well as 1,2-diketones gave no O-addition products under the current reaction conditions, likely caused by the intramolecular H-bonding.

## Conclusion

In this letter, we report the first successful gold(I)-catalyzed intermolecular O-vinylation of cyclic 1,3-diketones with unactivated alkynes. The reaction tolerates a large scope of alkynes, giving the desired O-addition products in good to excellent yields. The triazole coordinated gold catalysts gave improved reactivity compared with the typical [L-Au]^+^ by overcoming the undesired hydration. This discovery will likely benefit many future developments that currently suffer from the common hydration side reaction. The application of copper(II) triflate as the effective additive not only improves the reactivity, but also provides another example for plausible bimetallic catalysis, which is a very active research area in the gold catalysis community. Evaluation of distinct O-nucleophiles toward intermolecular addition to alkynes is currently underway in our group.

## Supporting Information

File 1 General methods, characterization data and NMR spectra of synthesized compounds.
