# Supplementary material for: Ambient gold-catalyzed O-vinylation of cyclic 1,3-diketone: A vinyl ether synthesis
Source: Beilstein J Org Chem. 2013 Nov 18;9:2537–43. doi: 10.3762/bjoc.9.288 (PMC3869262; doi:10.3762/bjoc.9.288)

Supporting Information

for

Ambient gold-catalyzed *O*-vinylation of cyclic 1,3-diketone: A vinyl ether synthesis

Yumeng Xi, Boliang Dong and Xiaodong Shi*

Address: C. Eugene Bennett Department of Chemistry, West Virginia University, Morgantown, West Virginia 26506, United States

Email: Xiaodong Shi* - xiaodong.shi@mail.wvu.edu

* Corresponding author

General methods, characterization data and NMR spectra of synthesized compounds

Table of Contents

I. General Methods and Materials S2
II. Compounds Characterization S3–S6
III. ORTEP Drawing of the Crystal Structure S7
IV. Reference S7
V. NMR Spectra of New Compounds S8–S25

I. General Methods and Materials

All of the reactions dealing with air and/or moisture-sensitive reactions were carried out under an atmosphere of nitrogen using oven/flame-dried glassware. Unless otherwise noted, all commercial reagents and solvents were obtained from the commercial provider and used without further purification. All gold complexes were synthesized from $HAuCl_4$, which was purchased from Strem. XPhos was purchased from Acros and used as received (stored at 4°C and handled in glovebox). ^{1}H NMR and ^{13}C NMR spectra were recorded on Varian/Agilent 400 MHz spectrometers. Chemical shifts were reported relative to internal tetramethylsilane (δ 0.00 ppm) or $CDCl_3$ (δ 7.26 ppm) for ^{1}H and $CDCl_3$ (δ 77.0 ppm) for ^{13}C. Flash column chromatography was performed on 230-430 mesh silica gel. HRMS were recorded on LTQ-FTUHRA spectrometer.

Representative procedure for Gold-catalyzed O-Vinylation of 1,3-Cyclic Diketone

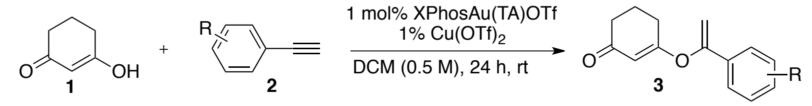

A 4 mL screw-cap vial was charged with 1,3-cyclohexanedione (46 mg, 0.4 mmol) and alkyne (0.8 mmol, 1.2 equiv.) in dry DCM (0.8 mL), followed by the addition of catalysts XPhosAu(TA)OTf (3.7 mg, 1 mol%) and $Cu(OTf)_2$ (1.4 mg, 1 mol%). The vial was allowed to stir at rt and monitored by TLC. After the reaction was completed (about 24 h), the reaction mixture was directly purified by flash chromatography on silica gel (ethyl acetate/hexane = 1:3, V/V) to give desired addition product.

Procedure for Synthesis of XPhosAu(TA)OTf

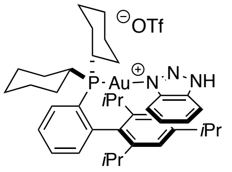

XPhosAu(TA)OTf

A 20 mL screw-cap vial was charged with XPhosAuCl (355 mg, 0.5 mmol) and 1H-benzotriazole (1.1 equiv.) in dry DCM (5 mL), followed by the addition of AgOTf (1.05 equiv.). The vial was allowed to stir at ambient temperature. After 4h, the reaction mixture was filtered through two celite pads and concentrated *in vacuo* to give the product as white foam. Pure white powder was obtained via recrystallization through diffusion of hexane into DCM solution of crude product.

For the synthesis of $Ph_3PAu(TA)OTf$ and IPrAu(TA)OTf, see: ref 1-2.

II. Compounds Characterizaton

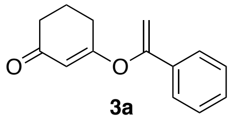

3a

^{1}H-NMR (400 MHz, $CDCl_3$) δ 7.42-7.40 (m, 2H), 7.33-7.30 (m, 2H), 5.43 (d, *J* = 2.0 Hz, 1H), 5.42 (s, 1H), 4.97 (d, *J* = 2.4 Hz, 1H), 2.61 (t, *J* = 6.4 Hz, 2H), 2.31 (t, *J* = 6.8 Hz, 2H), 2.61 (quint, *J* = 6.4 Hz, 2H), 2.00 (s, 6H).

^{13}C-NMR (100 MHz, $CDCl_3$) δ 199.6, 176.5, 154.9, 133.2, 129.3, 128.7, 125.0, 106.5, 101.9, 36.5, 28.2, 21.1.

HRMS Calculated for $C_{14}H_{15}O_2$ $[M+H]^+$: 215.1067, Found: 215.1067.

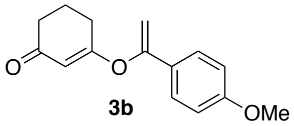

3b

^{1}H-NMR (400 MHz, $CDCl_3$) δ7.37-7.35 (m, 2H), δ6.87-6.84 (m, 2H), 5.31 (t, *J* = 1.6 Hz, 1H), 4.87 (d, *J* = 1.6 Hz, 1H), 3.80 (s, 3H), 2.63 (t, *J* = 1.6 Hz, 2H), 2.34 (t, *J* = 1.6 Hz, 2H), 2.05-2.02 (m, 2H).

^{13}C-NMR (100 MHz, $CDCl_3$) δ 199.7, 176.7, 160.3, 154.6, 126.4, 125.6, 114.0, 106.3, 99.9, 55.2, 36.4, 28.1, 21.0.

HRMS Calculated for $C_{15}H_{17}O_3$ $[M+H]^+$: 245.1172, Found: 245.1172.

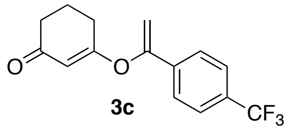

3c

^{1}H-NMR (400 MHz, $CDCl_3$) δ 7.61 (d, *J* = 8.0 Hz, 2H), 7.55 (d, *J* = 8.4 Hz, 2H), 5.57 (d, *J* = 2.4 Hz, 1H), 5.39 (s, 1H), 5.13 (d, *J* = 2.0 Hz, 1H), 2.64 (t, *J* = 6.2 Hz, 2H), 2.34 (t, *J* = 6.6 Hz, 2H), 2.06 (quint, *J* = 6.4 Hz, 2H).

^{13}C-NMR (100 MHz, $CDCl_3$) δ 199.3, 176.0, 153.5, 136.6, 126.0, 125.7 (q, *J* = 3.8 Hz), 125.3, 104.6, 104.1, 36.4, 28.1, 21.0.

HRMS Calculated for $C_{15}H_{14}F_3O_2$ $[M+H]^+$: 283.0940, Found: 283.0942.

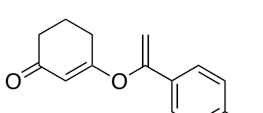

3d

^{1}H-NMR (400 MHz, $CDCl_3$) δ 7.43-7.39 (m, 2H), 7.06-7.00 (m, 2H), 7.35 (t, *J* = 7.5 Hz, 2H), 5.41 (s, 1H), 5.38 (d, *J* = 2.4 Hz, 1H), 4.97 (d, *J* = 2.4 Hz, 1H), 2.61 (t, *J* = 6.4 Hz, 2H), 2.33 (t, *J* = 6.8 Hz, 2H), 2.04 (quint, *J* = 6.8 Hz, 2H).

^{13}C-NMR (100 MHz, $CDCl_3$) δ 199.3, 176.2, 164.4, 161.9, 153.9, 129.4, 126.9, 126.9, 115.6 (q, *J* = 22.0 Hz), 106.4, 101.6, 36.4, 28.1, 21.0.

HRMS Calculated for $C_{14}H_{14}FO_2$ $[M+H]^+$: 233.0972, Found: 233.0971.

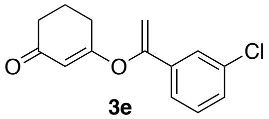

3e

^{1}H-NMR (400 MHz, $CDCl_3$) δ 7.41-7.40 (m, 1H), 7.34-7.27 (m, 3H), 5.47 (d, *J* = 2.4 Hz, 1H), 5.04 (d, *J* = 2.4 Hz, 1H), 2.63 (t, *J* = 6.2 Hz, 2H), 2.34 (t, *J* = 6.6 Hz, 2H), 2.05 (quint, *J* = 6.4 Hz, 2H).

^{13}C-NMR (100 MHz, $CDCl_3$) δ 199.4, 176.1, 153.5, 135.1, 134.7, 130.0, 129.3, 125.1, 123.2, 106.6, 103.2, 36.5, 28.1, 21.0.

HRMS Calculated for $C_{14}H_{14}ClO_2$ $[M+H]^+$: 249.0677, Found: 249.0678.

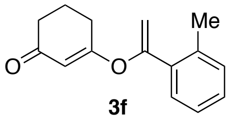

3f

^{1}H-NMR (400 MHz, $CDCl_3$) δ 7.36-7.34 (m, 1H), 7.26-7.21 (m, 1H), 7.18-7.14 (m, 2H), 5.44 (s, 1H), 5.15 (d, *J* = 2.0 Hz, 1H), 5.06 (d, *J* = 1.6 Hz, 1H), 2.50 (t, *J* = 6.4 Hz, 2H), 2.41 (s, 3H), 2.27 (t, *J* = 6.4 Hz, 2H), 1.97 (quint, *J* = 6.4 Hz, 2H).

^{13}C-NMR (100 MHz, $CDCl_3$) δ 199.3, 175.5, 155.6, 135.8, 133.8, 130.9, 129.0,128.9, 125.8, 106.7, 106.5, 36.4, 28.3, 210.0, 20.7.

HRMS Calculated for $C_{15}H_{17}O_2$ $[M+H]^+$: 229.1223, Found: 229.1224.

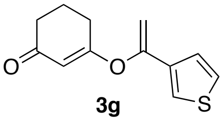

3g

^{1}H-NMR (400 MHz, $CDCl_3$) δ 7.29-7.25 (m, 2H), 7.14 (dd, *J* = 5.0, 1.4 Hz, 1H), 5.49 (s, 1H), 5.32 (d, *J* = 2.0 Hz, 1H), 4.91 (d, *J* = 2.0 Hz, 1H), 2.61 (t, *J* = 6.4 Hz, 2H), 2.34 (t, *J* = 6.4 Hz, 2H), 2.04 (quint, *J* = 6.4 Hz, 2H).

^{13}C-NMR (100 MHz, $CDCl_3$) δ 199.5, 176.4, 151.0, 135.3, 126.6, 124.7, 122.4, 106.1, 101.1, 36.4, 28.1, 21.0.

HRMS Calculated for $C_{12}H_{13}O_2S$ $[M+H]^+$: 221.0631, Found: 221.0631.

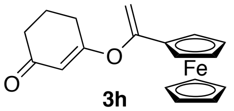

3h

^{1}H-NMR (400 MHz, $CDCl_3$) δ 5.56 (s, 1H), 5.11 (d, *J* = 2.0 Hz, 1H), 4.75 (d, *J* = 2.0 Hz, 1H), 4.31 (t, *J* = 1.8 Hz, 2H), 4.22 (t, *J* = 1.8 Hz, 2H), 4.13 (s, 5H), 2.58 (t, *J* = 6.2 Hz, 2H), 2.35 (t, *J* = 6.2 Hz, 2H), 2.04 (quint, *J* = 6.4 Hz, 2H).

^{13}C-NMR (100 MHz, $CDCl_3$) δ 199.5, 176.6, 155.2, 105.8, 98.6, 78.4, 69.7, 69.2, 66.3, 36.6, 28.2, 21.1.

HRMS Calculated for $C_{18}H_{19}FeO_2$ $[M+H]^+$: 323.0734, Found: 321.0729.

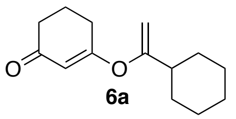

6a

^{1}H-NMR (400 MHz, $CDCl_3$) δ 5.41 (s, 1H), 4.64 (m, 1H), 4.51 (m, 1H), 2.41 (t, *J* = 6.3 Hz, 2H), 2.26 (t, *J* = 6.8 Hz, 2H), 1.98-1.90 (m, 3H), 1.79-1.57 (m, 4H), 1.17-1.04 (m, 6H).

^{13}C-NMR (100 MHz, $CDCl_3$) δ 199.6, 176.7, 162.2, 105.2, 99.2, 40.7, 36.4, 30.5, 28.3, 25.8, 25.7, 21.0.

HRMS Calculated for $C_{14}H_{21}O_2$ $[M+H]^+$: 221.1536, Found: 221.1535.

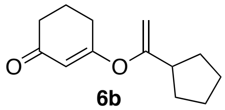

6b

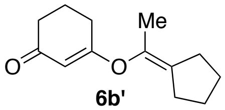

6b'

Obtained as inseparable mixtures (**6b:6b'**=1:0.4).

Major isomer (6b):

^{1}H-NMR (400 MHz, $CDCl_3$) δ7.37-7.35 (m, 2H), δ 5.56 (s, 1H), 4.80 (dd, *J* = 1.6, 1.0 Hz, 1H), 4.60 (d, *J* = 1.6 Hz, 1H), 2.59 (quint, *J* = 8.0 Hz, 1H), 2.50 (t, *J* = 6.4 Hz, 2H), 2.36 (t, *J* = 6.4 Hz, 2H), 1.84-1.80 (m, 2H), 1.71-1.47 (m, 6H).

^{13}C-NMR (100 MHz, $CDCl_3$) δ 199.8, 176.8, 161.0, 105.4, 99.5, 42.9, 36.6, 30.6, 28.4, 24.8, 21.1.

HRMS Calculated for $C_{13}H_{19}O_2$ $[M+H]^+$: 207.1380, Found: 207.1378.

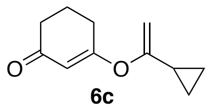

6c

^{1}H-NMR (400 MHz, $CDCl_3$) δ 5.60 (s, 1H), 4.75 (d, *J* = 2.0 Hz, 1H), 4.57 (d, *J* = 1.6 Hz, 1H), 2.48 (t, *J* = 6.8 Hz, 2H), 2.35 (t, *J* = 6.8 Hz, 2H), 2.02 (quint, *J* = 6.4 Hz, 2H), 1.49 (tt, *J* = 8.4, 5.2 Hz, 1H), 0.75-0.70 (m, 2H), 0.61-0.57 (m, 2H).

^{13}C-NMR (100 MHz, $CDCl_3$) δ 199.7, 176.7, 158.3, 105.6, 98.9, 36.5, 28.2, 21.1, 13.0, 6.0.

HRMS Calculated for $C_{11}H_{15}O_2$ $[M+H]^+$: 179.1067, Found: 179.1066.

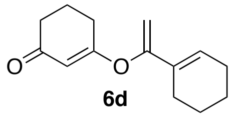

6d

^{1}H-NMR (400 MHz, $CDCl_3$) δ 5.91 (t, *J* = 5.4 Hz, 1H), 5.42 (s, 1H), 4.94 (d, *J* = 1.2 Hz, 1H), 4.69 (d, *J* = 0.8 Hz, 1H), 2.56 (t, *J* = 6.2 Hz, 2H), 2.35 (t, *J* = 6.4 Hz, 2H), 2.15-2.10 (m, 4H), 2.03 (quint, *J* = 6.6 Hz, 2H), 1.71-1.66 (m, 2H), 1.60-1.55 (m, 2H).

^{13}C-NMR (100 MHz, $CDCl_3$) δ 199.9, 177.5, 155.8, 129.0, 126.7, 105.2, 99.9, 36.5, 28.1, 25.5, 24.6, 22.1, 21.6, 21.1.

HRMS Calculated for $C_{14}H_{19}O_2$ $[M+H]^+$: 219.1380, Found: 219.1380.

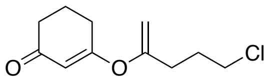

6e

^{1}H-NMR (400 MHz, $CDCl_3$) δ 5.45 (s, 1H), 4.78 (m, 1H), 4.68 (d, J = 1.6 Hz, 1H), 3.53 (t, J = 6.4 Hz, 2H), 2.44 (t, J = 6.0 Hz, 2H), 2.33-2.29 (m, 4H), 1.98 (quint, J = 6.4 Hz, 2H), 1.94-1.87 (m, 2H).
^{13}C-NMR (100 MHz, $CDCl_3$) δ 199.5, 175.8, 156.1, 105.5, 102.4, 43.7, 36.5, 29.4, 29.3, 28.3, 21.0.
HRMS Calculated for $C_{11}H_{16}ClO_2$ $[M+H]^+$: 215.0833, Found: 215.0832.

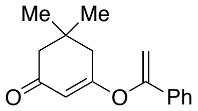

10a

^{1}H-NMR (400 MHz, $CDCl_3$) δ 7.43-7.41 (m, 2H), 7.35-7.32 (m, 3H), 5.44 (t, J = 2.4 Hz, 1H), 5.42 (s, 1H), 4.98 (d, J = 2.0 Hz, 1H), 2.49 (s, 2H), 2.19 (s, 2H), 1.10 (s, 2H).
^{13}C-NMR (100 MHz, $CDCl_3$) δ 199.4, 175.0, 155.0, 133.2, 129.3, 128.7, 125.0, 105.4, 101.9, 50.6, 42.1, 32.7, 28.2.
HRMS Calculated for $C_{16}H_{18}O_2Na$ $[M+H]^+$: 265.1199, Found: 265.1199.

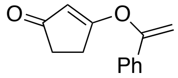

10b

^{1}H-NMR (400 MHz, $CDCl_3$) δ 7.44-7.41 (m, 2H), 7.34-7.31 (m, 3H), 5.42 (d, J = 2.8 Hz, 1H), 5.26 (t, J = 1.2 Hz, 1H), 5.04 (d, J = 2.4 Hz, 1H), 2.77-2.74 (m, 2H), 2.47-2.44 (m, 2H).
^{13}C-NMR (100 MHz, $CDCl_3$) δ 205.5, 188.7, 156.8, 132.6, 129.4, 128.7, 125.0, 108.4, 101.1, 34.3, 28.0.
HRMS Calculated for $C_{13}H_{13}O_2$ $[M+H]^+$: 201.0910, Found: 201.0909.

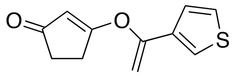

10c

^{1}H-NMR (400 MHz, $CDCl_3$) δ 7.33-7.31 (m, 2H), 7.17 (d, J = 5.4, 2.4 Hz, 1H), 5.37 (t, J = 0.9 Hz, 1H), 5.33 (d, J = 2.4 Hz, 1H), 5.00 (d, J = 2.4 Hz, 1H), 2.81-2.78 (m, 2H), 2.53-2.50 (m, 2H).
^{13}C-NMR (100 MHz, $CDCl_3$) δ 205.5, 188.7, 153.1, 134.8, 126.9, 124.7, 122.7, 108.1, 100.2, 34.4, 28.0.
HRMS Calculated for $C_{11}H_{11}O_2$ $[M+H]^+$: 207.0474, Found: 207.0473.

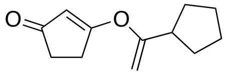

10d

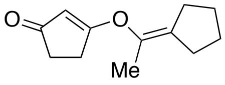

10d'

Obtained as inseparable mixtures (**10d:10d'**=1:2.6).
HRMS Calculated for $C_{12}H_{17}O_2$ $[M+H]^+$: 193.1223, Found: 193.1222.

III. ORTEP Drawing of the Crystal Structure

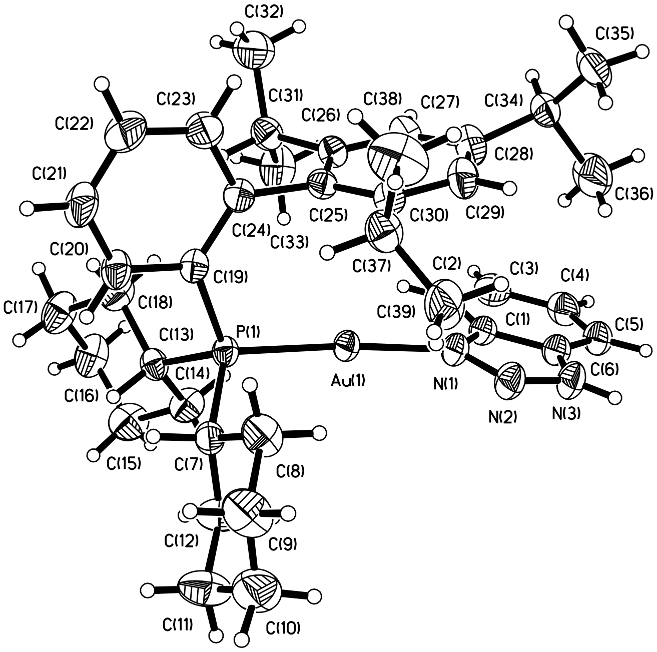

Figure S1. Perspective view of the molecular structure of the $(C_6H_5N_3)Au[P(C_6H_{11})_2(C_6H_4(C_6H_2(i\text{-}C_3H_7)_3))^+$ cation with the atom labeling scheme. The thermal ellipsoids are scaled to enclose 30% probability.

IV. Reference

1. Duan, H.; Sengupta, S.; Petersen, J. L.; Akhmedov, N.; Shi, X. *J. Am. Chem. Soc.* **2009,** *131*, 12100-12102.
2. Wang, D.; Cai, R.; Sharma, S.; Jirak, J.; Thummanapelli, S. K.; Akhmedov, N. G.; Zhang, H.;Liu, X.; Petersen, J. L.; Shi, X. *J. Am. Chem. Soc***.** **2012**, *134*, 9012-9019.

V. NMR Spectra of New Compounds

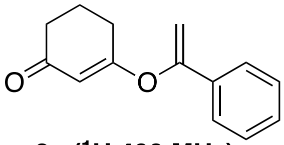

3a (^{1}H 400 MHz)

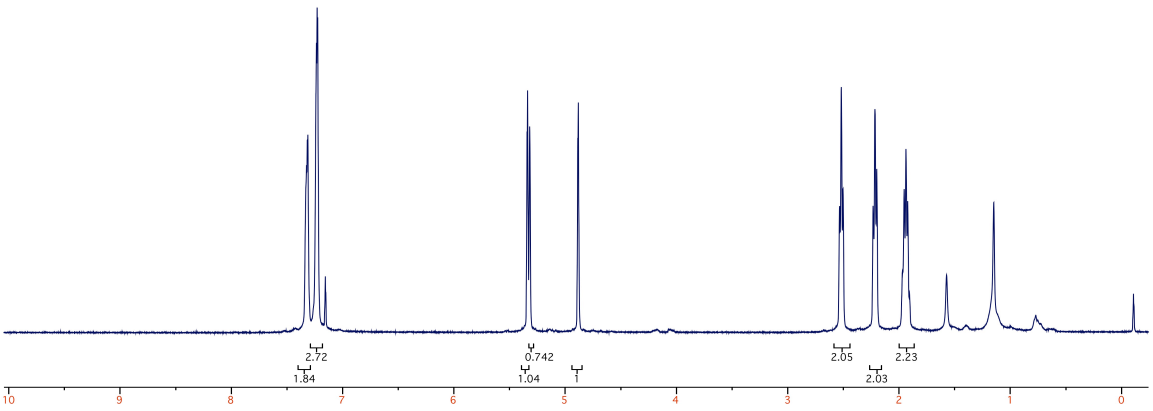

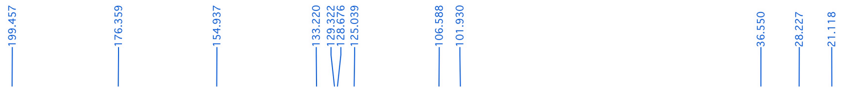

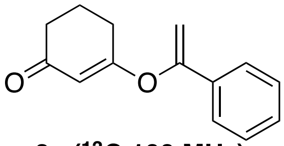

3a (^{13}C 100 MHz)

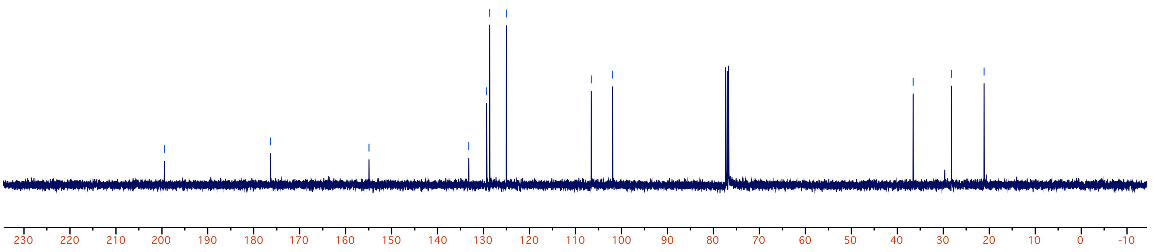

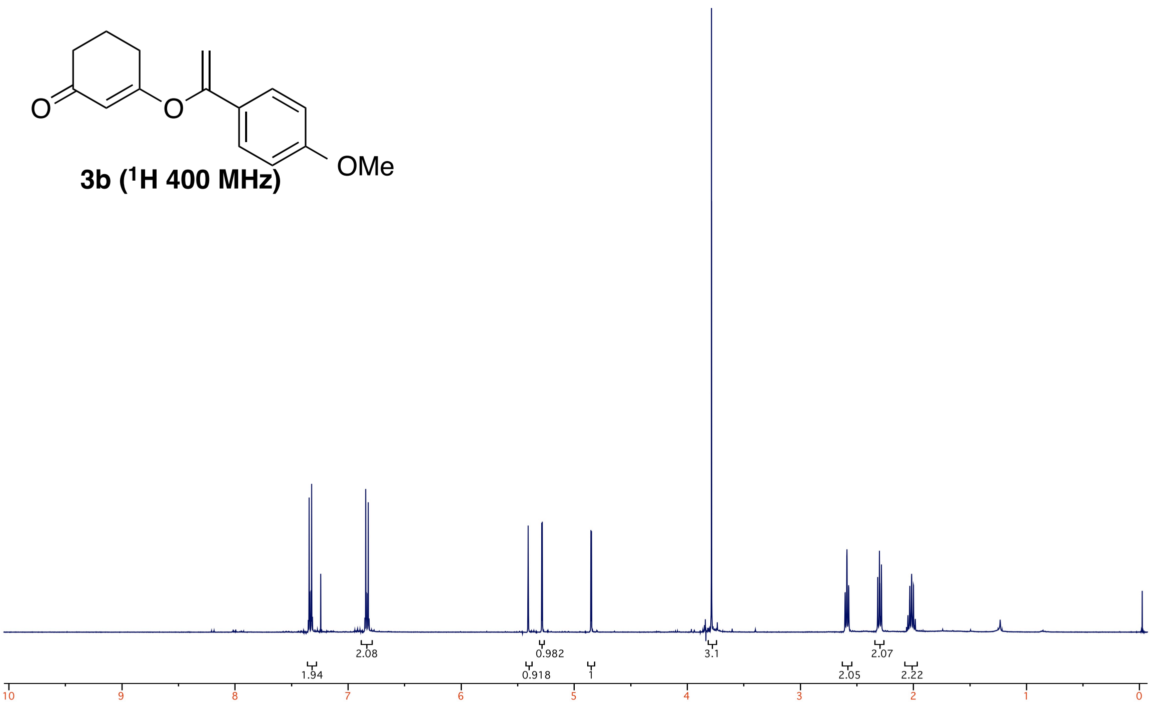
O
O
OMe
3b (1H 400 MHz)
2.08
0.982
3.1
2.07
1.94
0.918
1
2.05
2.22
10
9
8
7
6
5
4
3
2
1
0

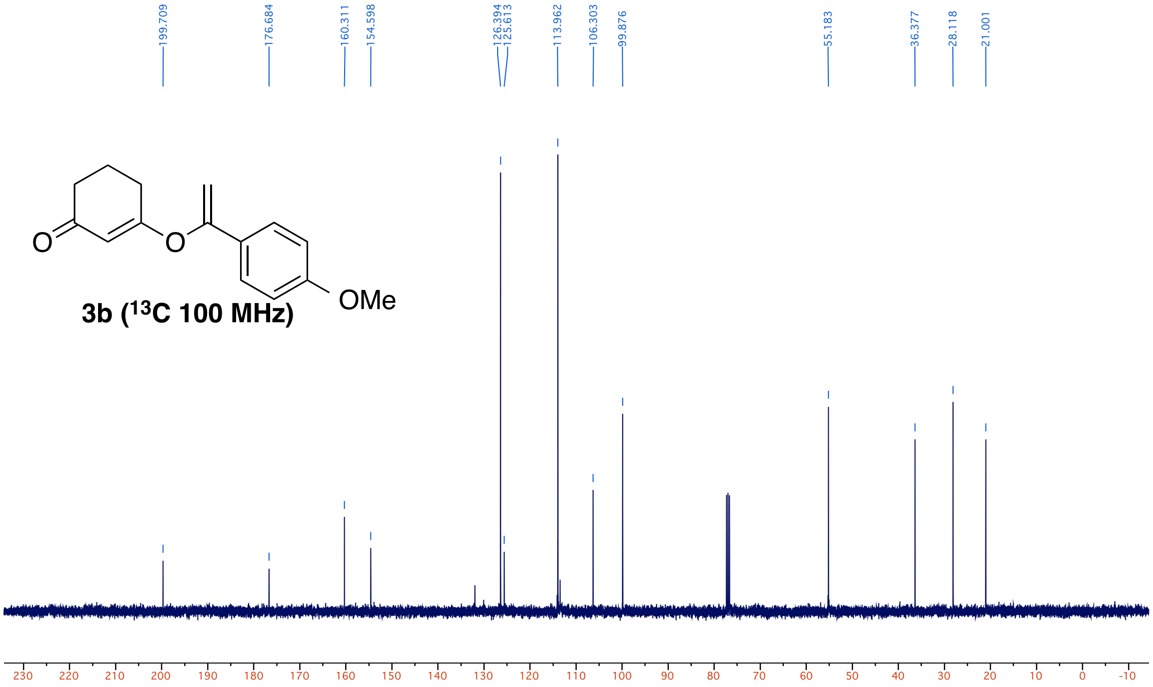
199.709
176.684
160.311
154.598
126.394
125.613
113.962
106.303
99.876
55.183
36.377
28.118
21.001
O
O
OMe
3b (13C 100 MHz)
230
220
210
200
190
180
170
160
150
140
130
120
110
100
90
80
70
60
50
40
30
20
10
0
-10

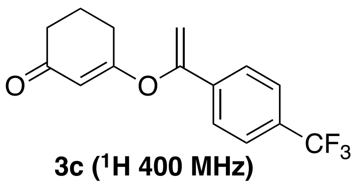

3c (^{1}H 400 MHz)

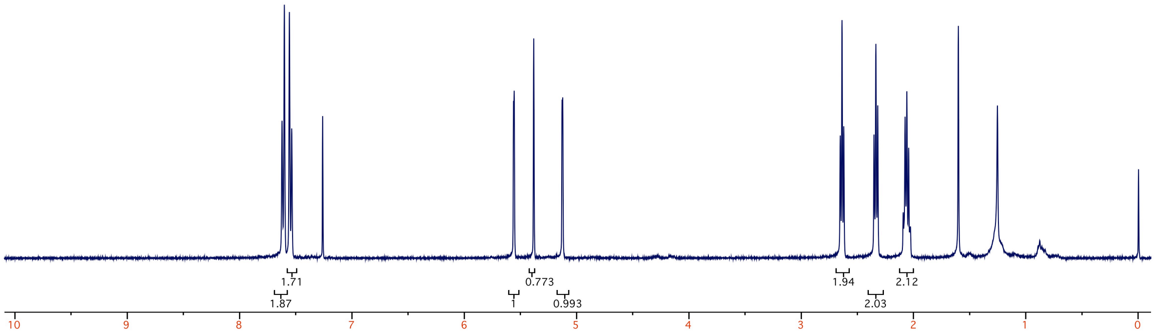

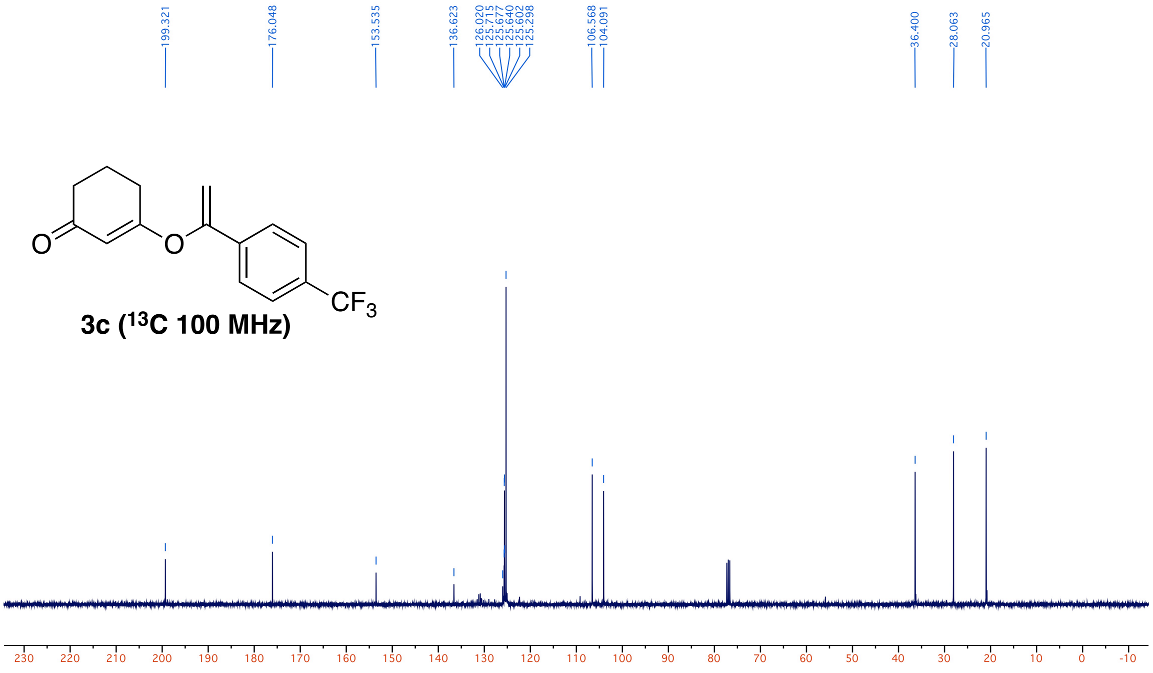

3c (^{13}C 100 MHz)

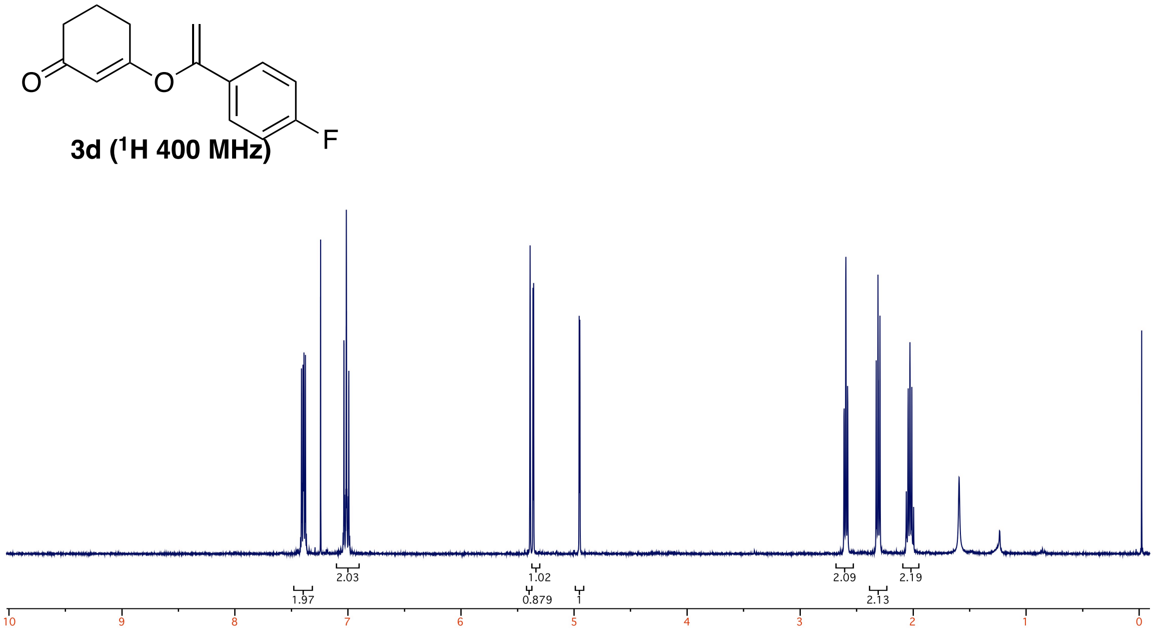

3d (^{1}H 400 MHz)

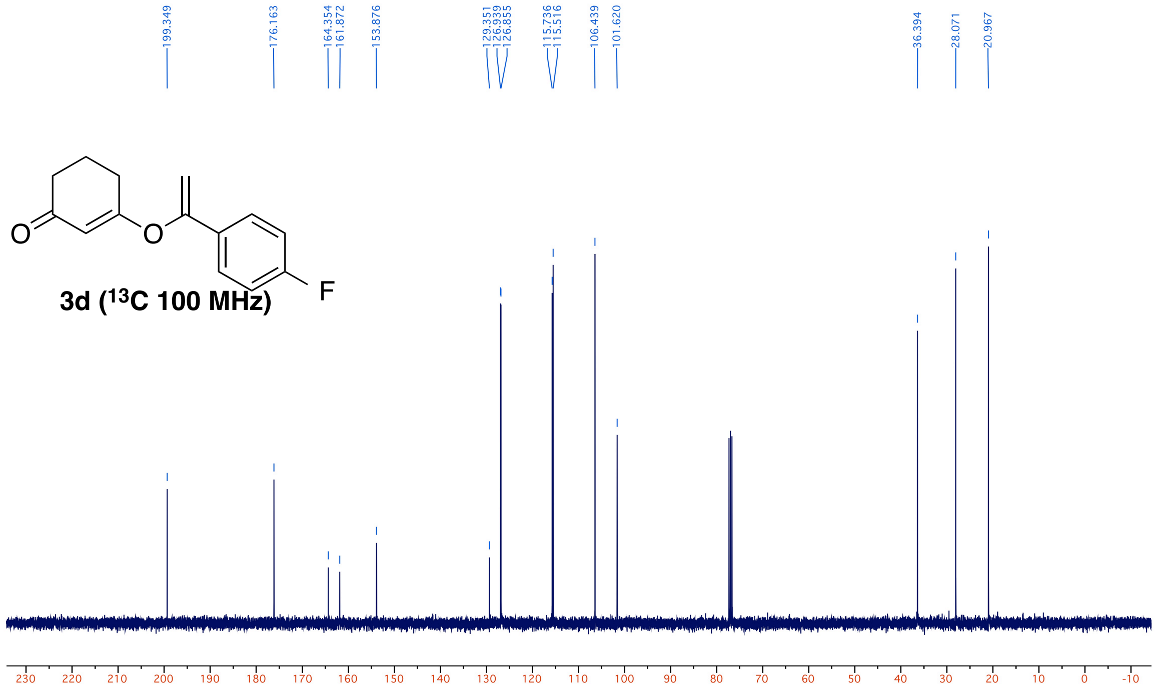

3d (^{13}C 100 MHz)

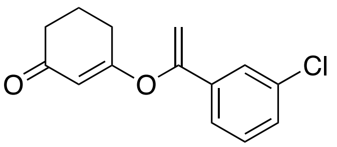

3e (¹H 400 MHz)

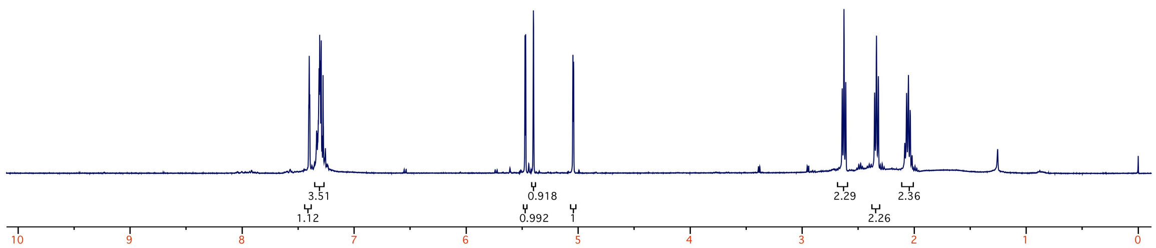

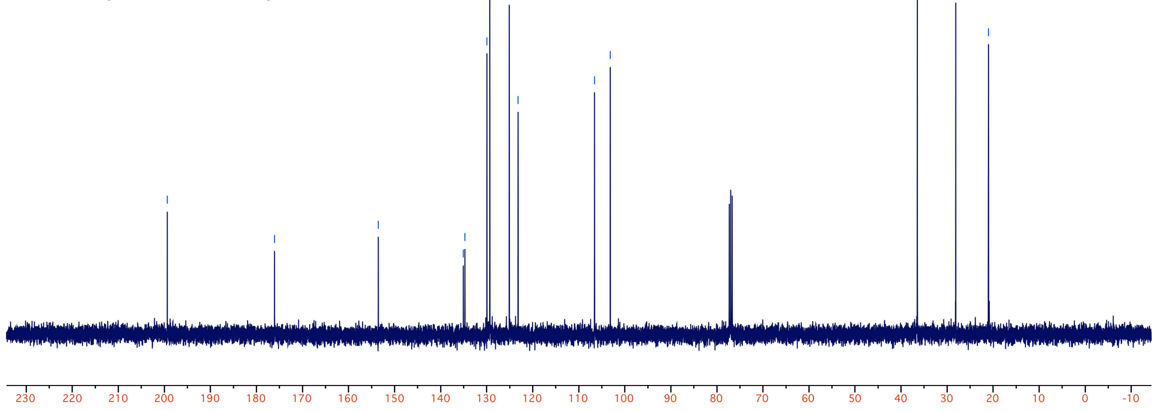

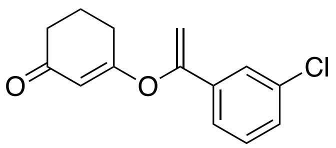

3e (¹³C 100 MHz)

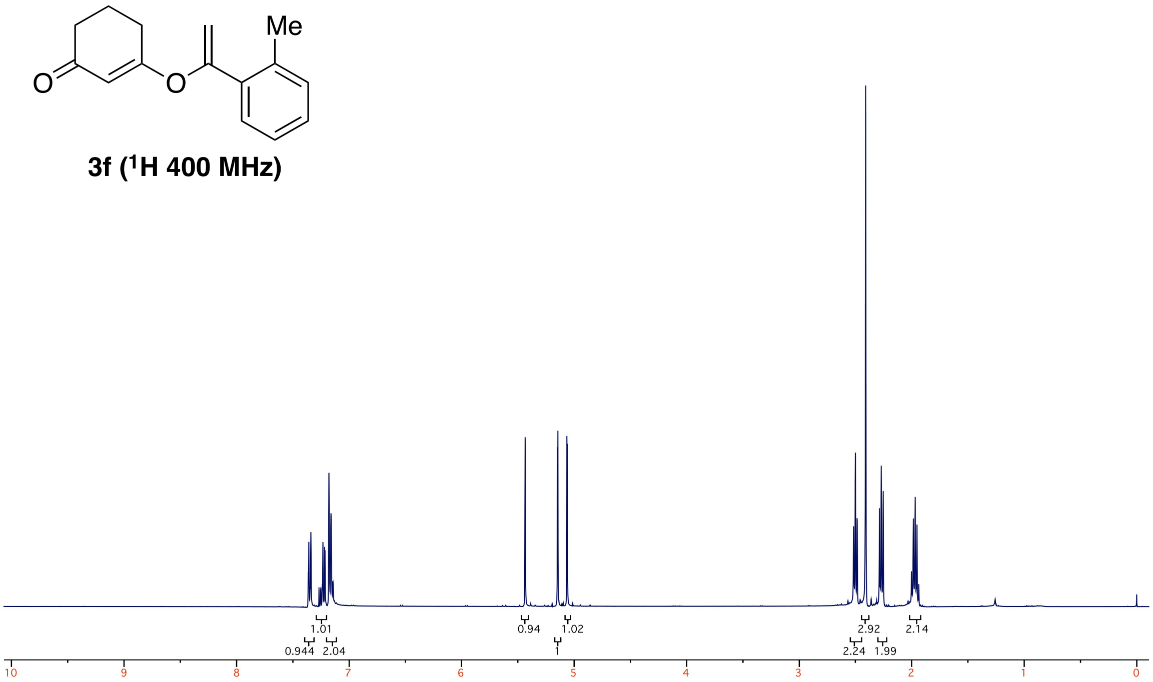
Me
O
O
3f (¹H 400 MHz)
1.01
0.944 2.04
0.94 1.02
1
2.92 2.14
2.24 1.99
10 9 8 7 6 5 4 3 2 1 0

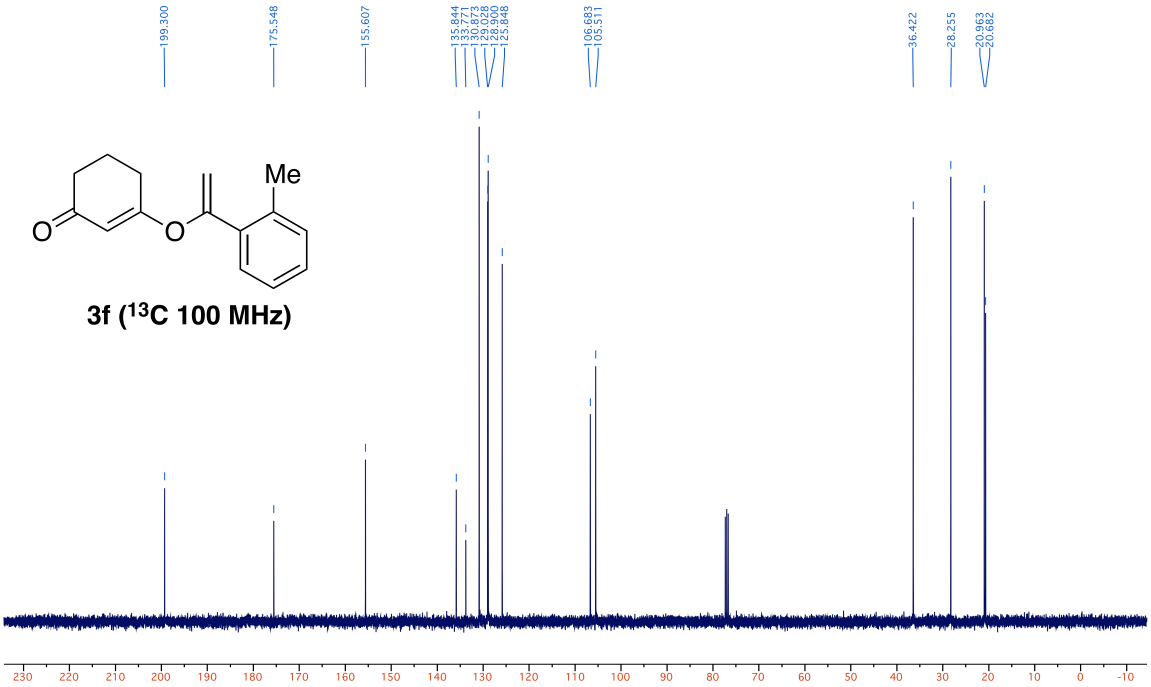
199.300
175.548
155.607
135.844
133.771
130.873
129.028
128.900
125.848
106.683
105.511
36.422
28.255
20.963
20.682
Me
O
O
3f (¹³C 100 MHz)
230 220 210 200 190 180 170 160 150 140 130 120 110 100 90 80 70 60 50 40 30 20 10 0 -10

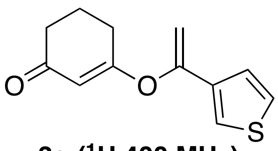

3g (^{1}H 400 MHz)

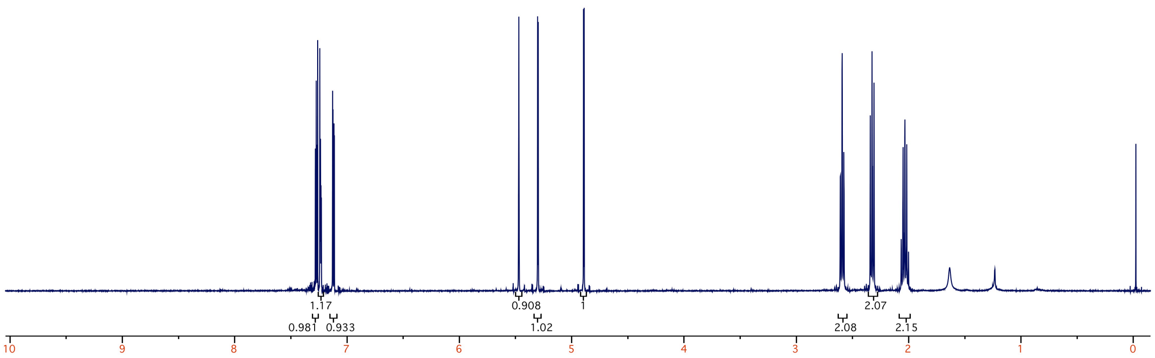

3g (^{13}C 100 MHz)

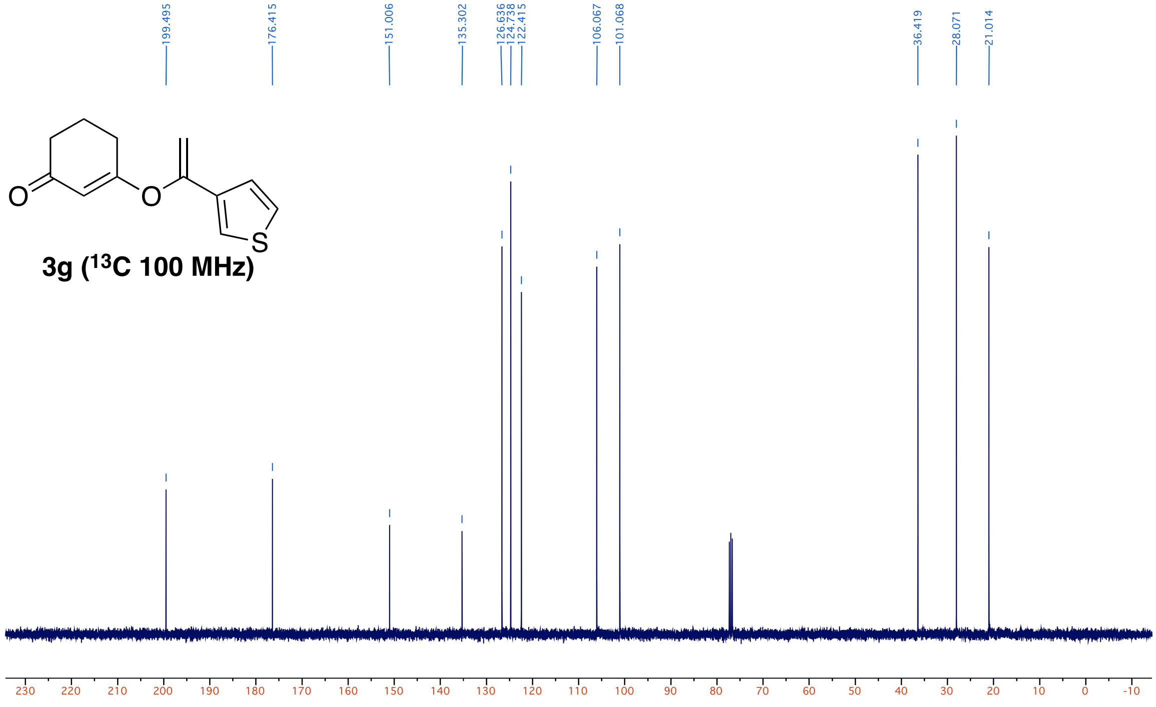

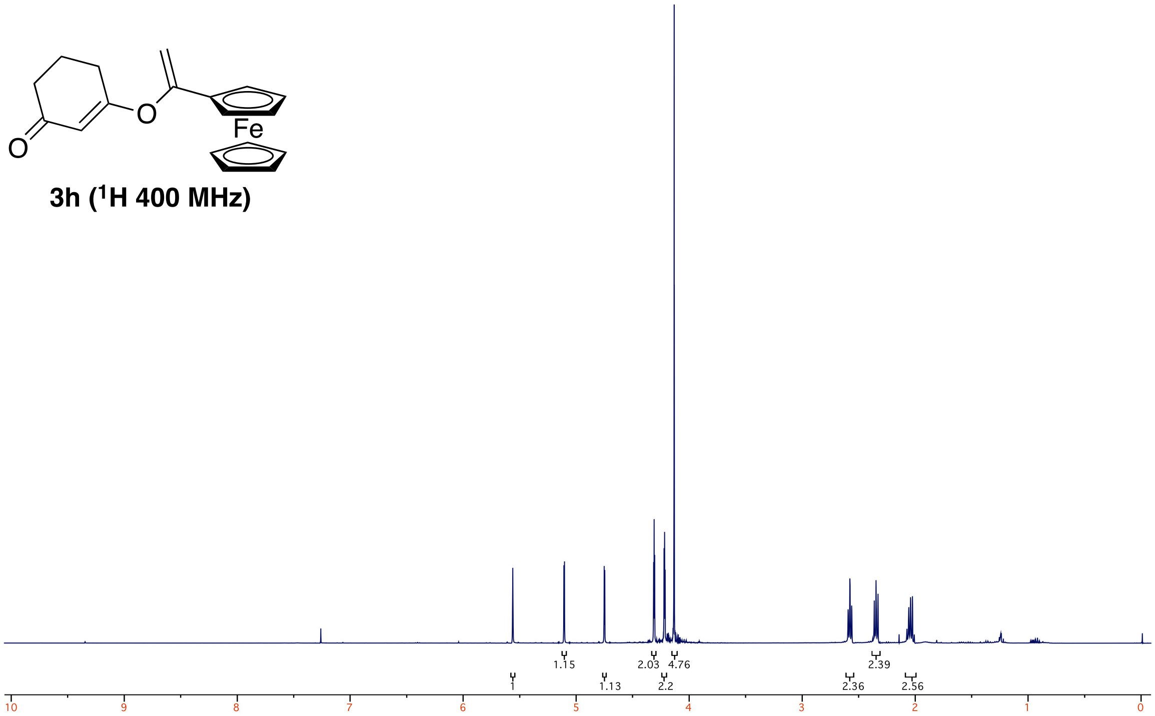

3h (^{1}H 400 MHz)

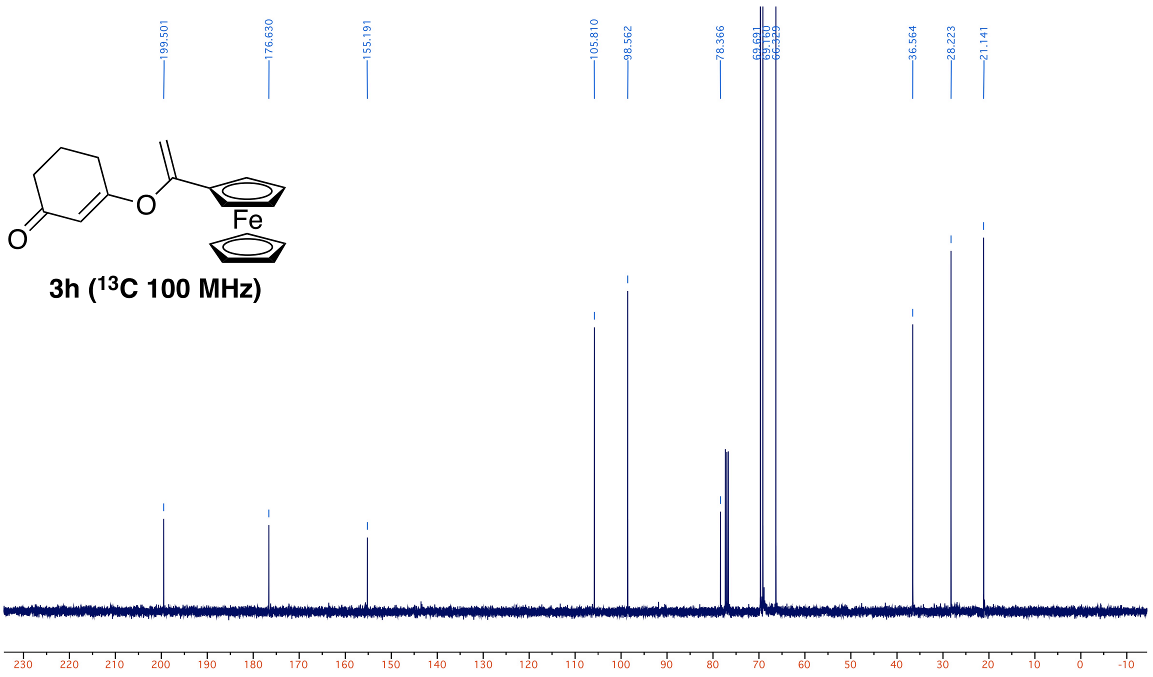

3h (^{13}C 100 MHz)

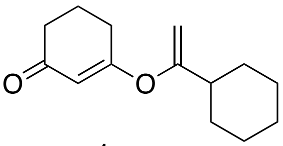

6a (^{1}H 400 MHz)

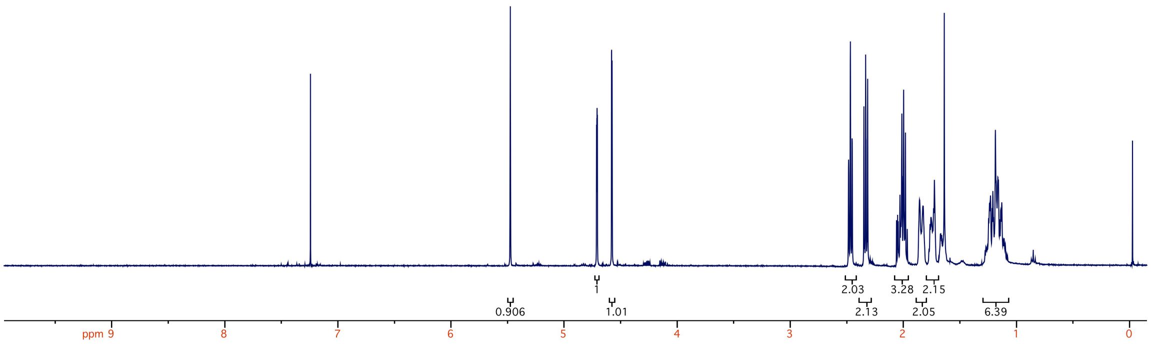

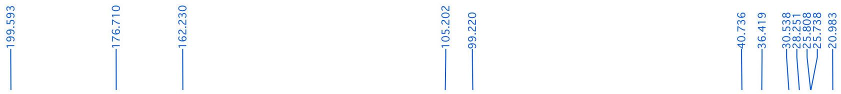

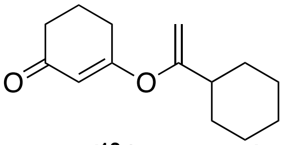

6a (^{13}C 100 MHz)

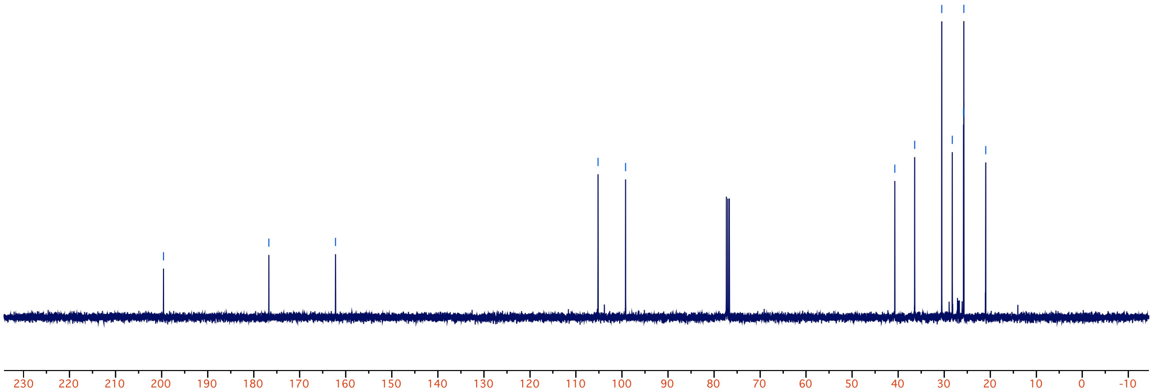

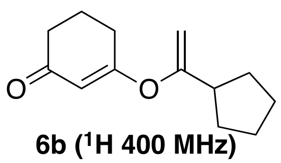

6b (^{1}H 400 MHz)

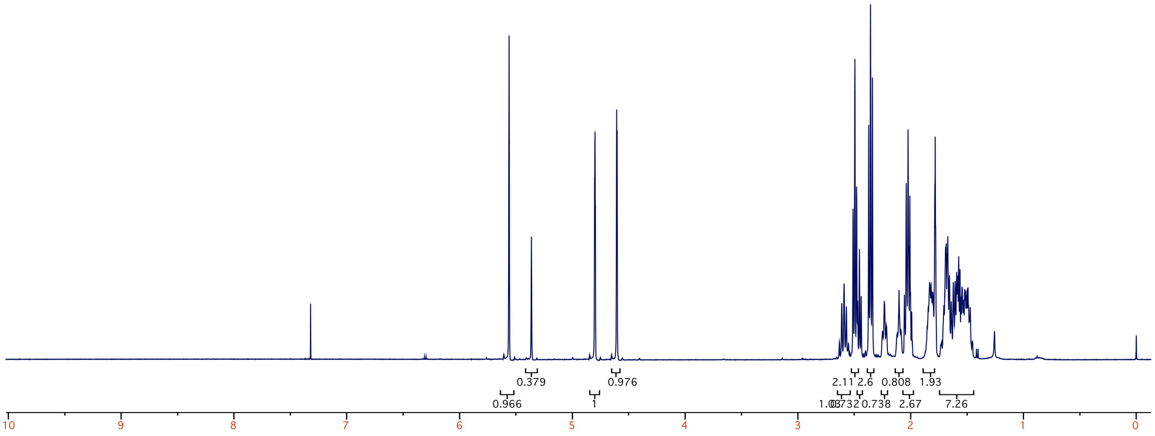

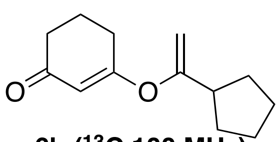

6b (^{13}C 100 MHz)

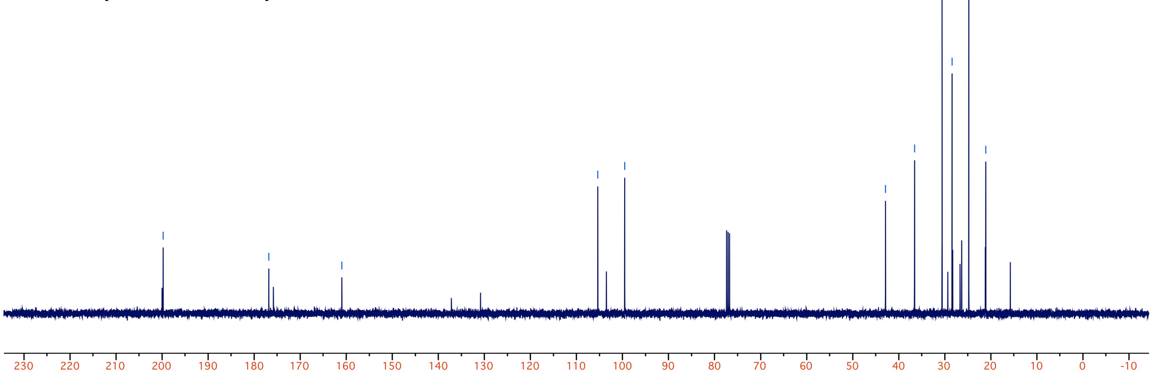

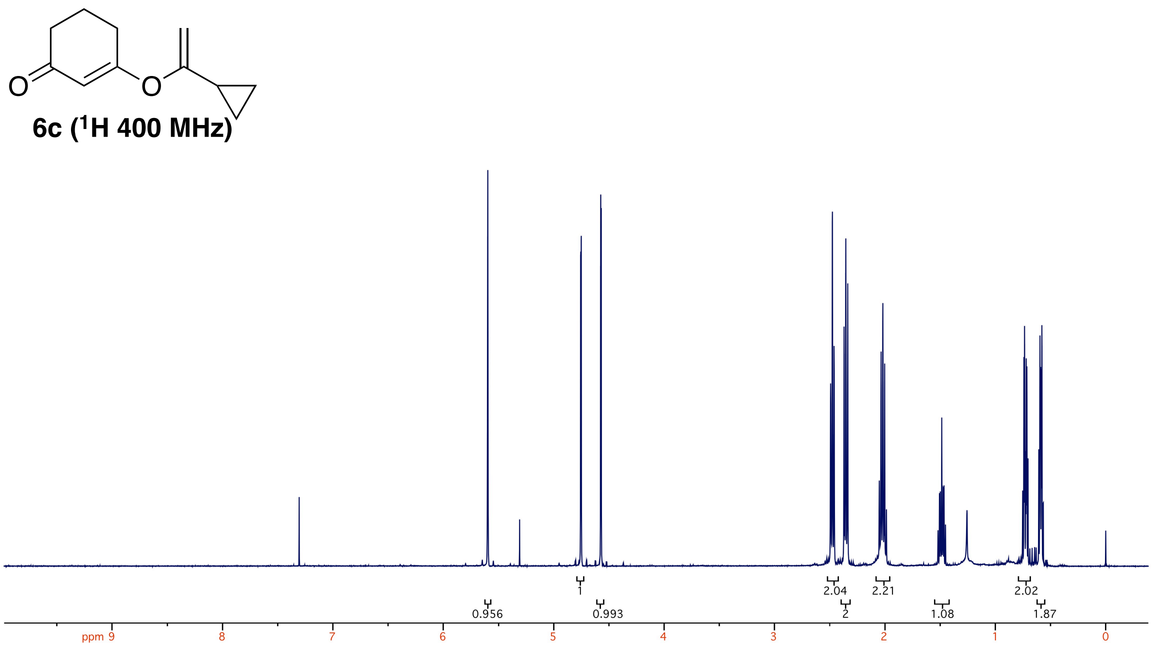
6c (1H 400 MHz)
0.956
1
0.993
2.04
2
2.21
1.08
2.02
1.87
ppm 9
8
7
6
5
4
3
2
1
0

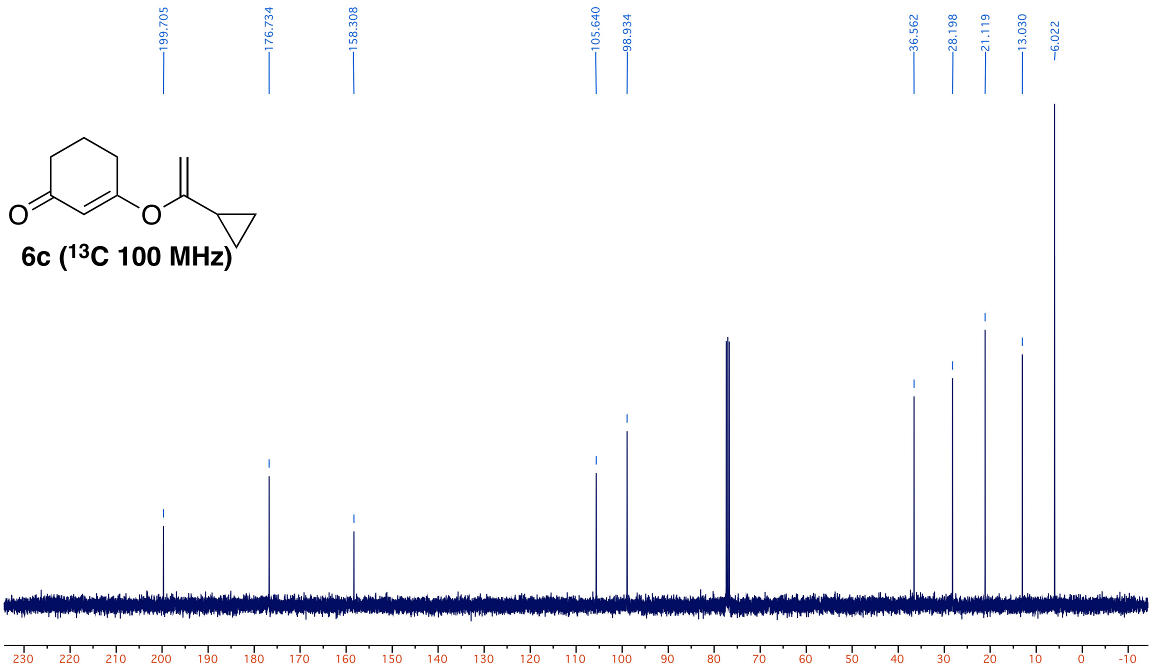
199.705
176.734
158.308
105.640
98.934
36.562
28.198
21.119
13.030
6.022
6c (13C 100 MHz)
230
220
210
200
190
180
170
160
150
140
130
120
110
100
90
80
70
60
50
40
30
20
10
0
-10

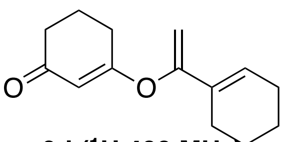

6d (^{1}H 400 MHz)

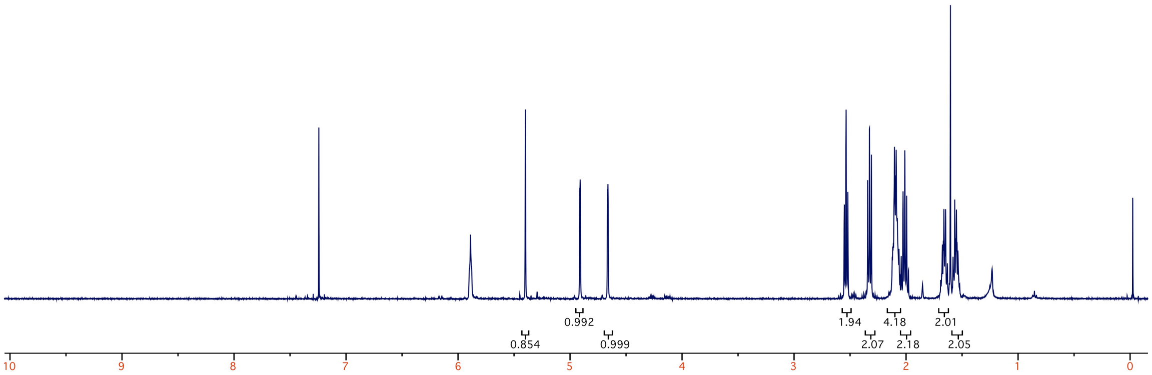

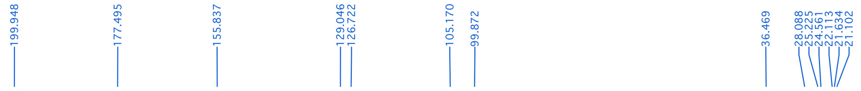

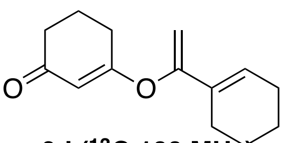

6d (^{13}C 100 MHz)

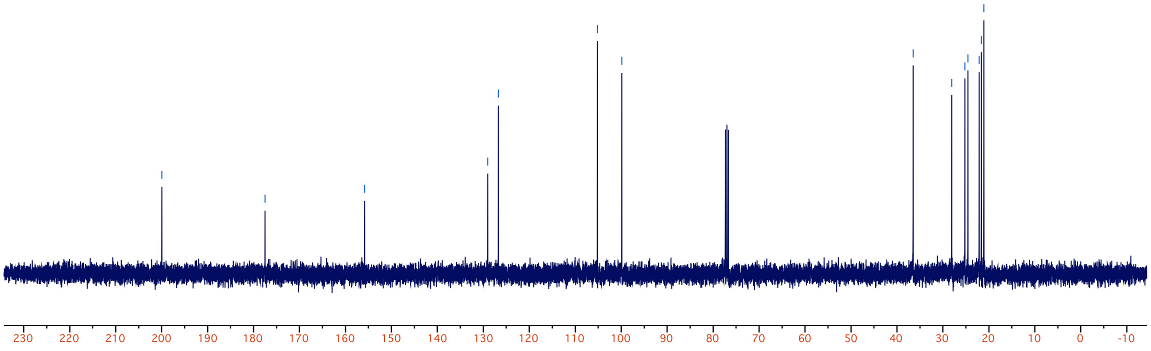

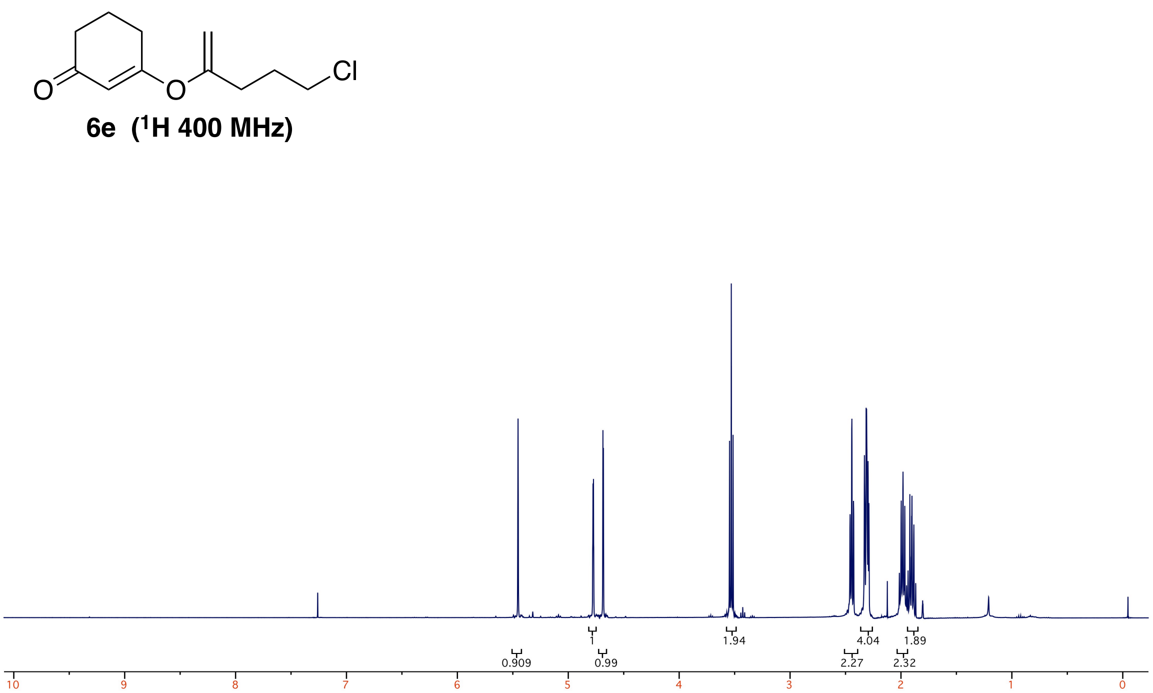
O
O
Cl
6e (¹H 400 MHz)
1
0.909
0.99
1.94
4.04
2.27
1.89
2.32
10
9
8
7
6
5
4
3
2
1
0

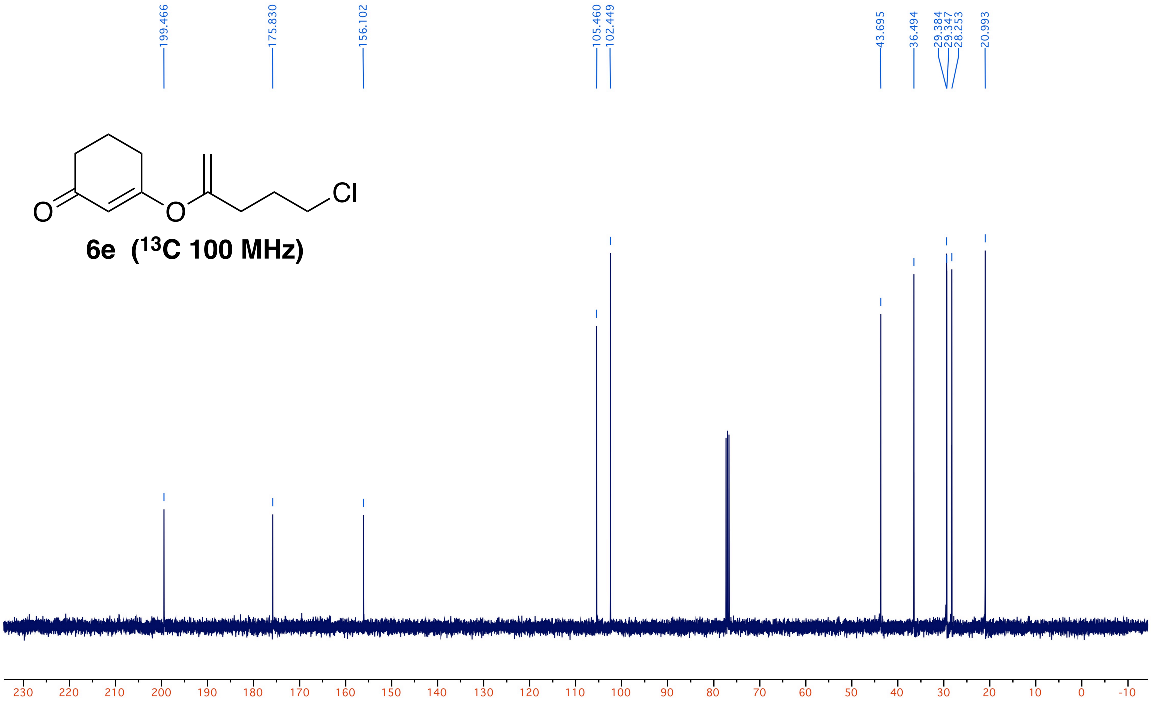
199.466
175.830
156.102
105.460
102.449
43.695
36.494
29.384
29.347
28.253
20.993
O
O
Cl
6e (¹³C 100 MHz)
230
220
210
200
190
180
170
160
150
140
130
120
110
100
90
80
70
60
50
40
30
20
10
0
-10

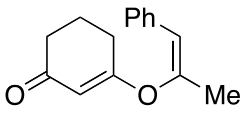

8a

(**^{1}H 400 MHz**)

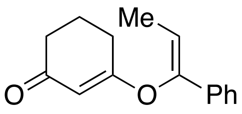

8b

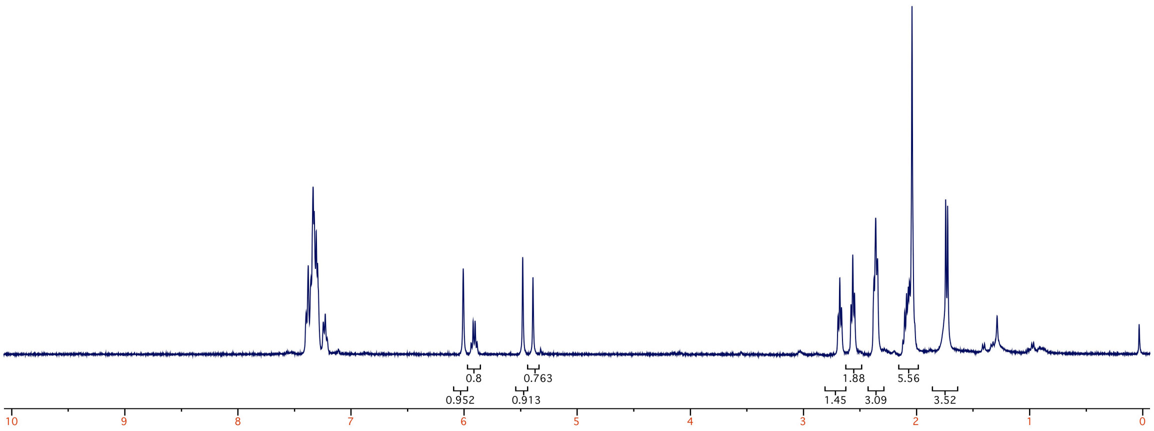

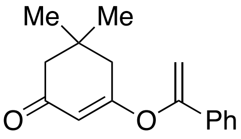

10a (^{1}H 400 MHz)

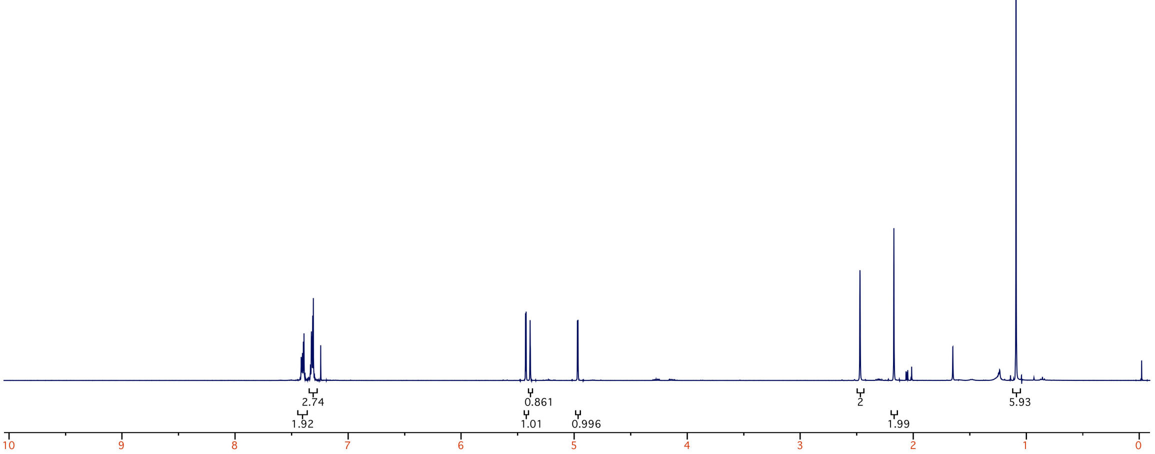

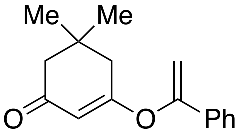

10a (^{13}C 100 MHz)

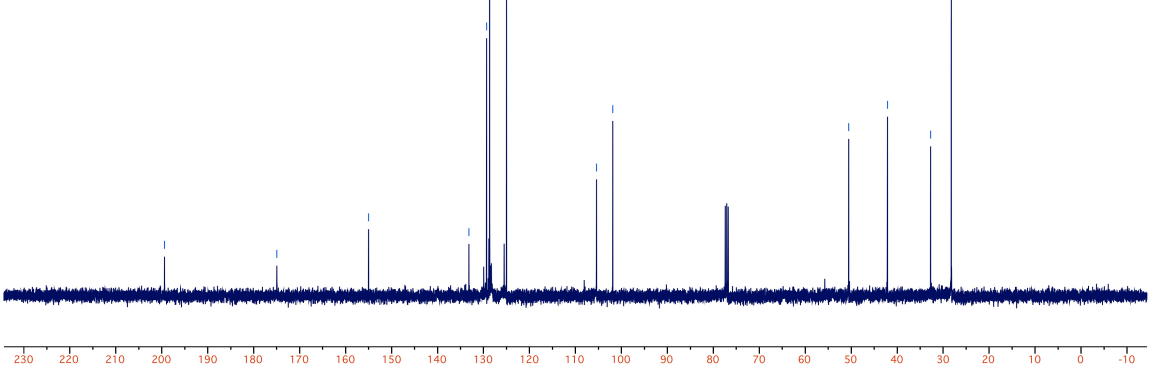

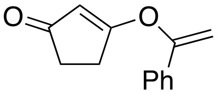

10b (^{1}H 400 MHz)

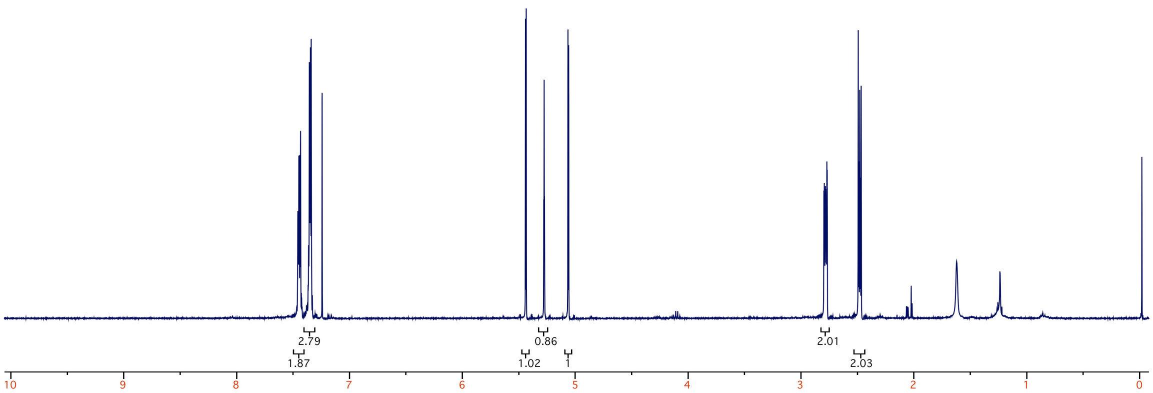

10b (^{13}C 100 MHz)

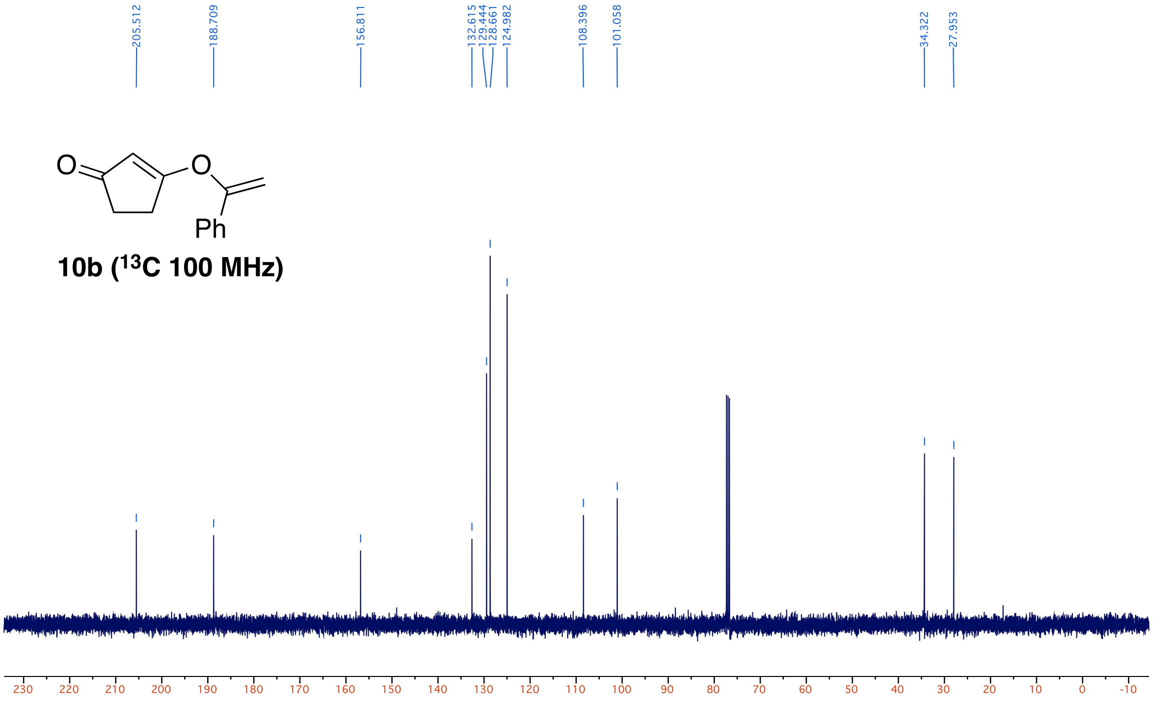

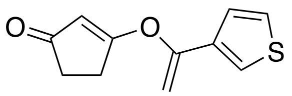

10c (¹H 400 MHz)

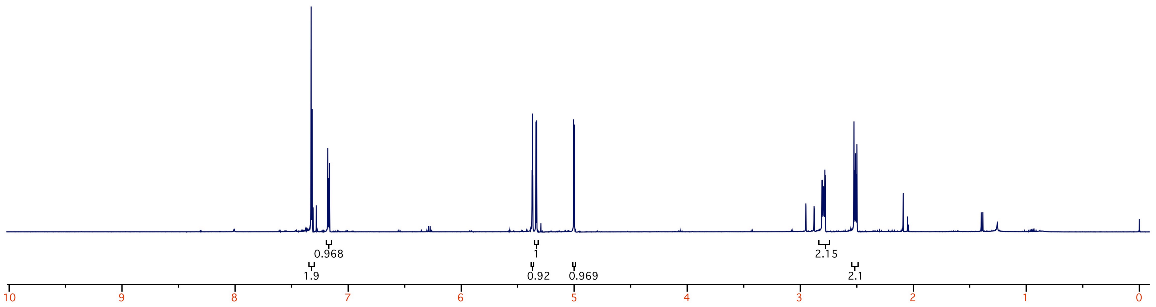

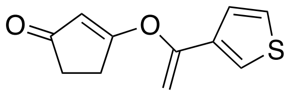

10c (¹H 400 MHz)

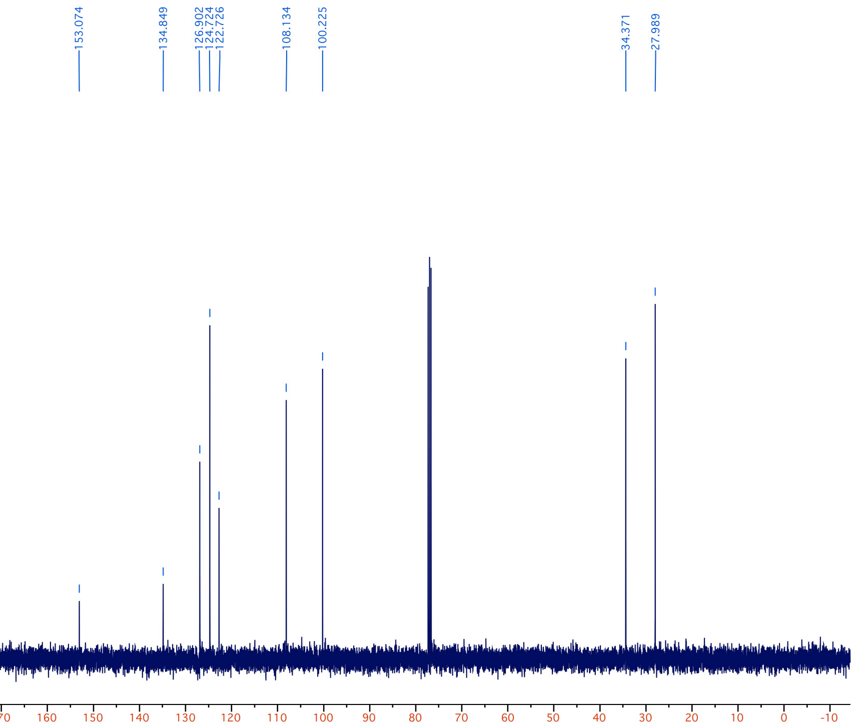

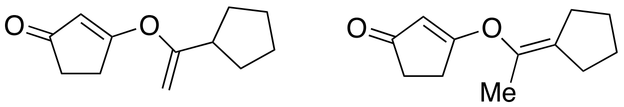

10d **(^{1}H 400 MHz)** **10d'**

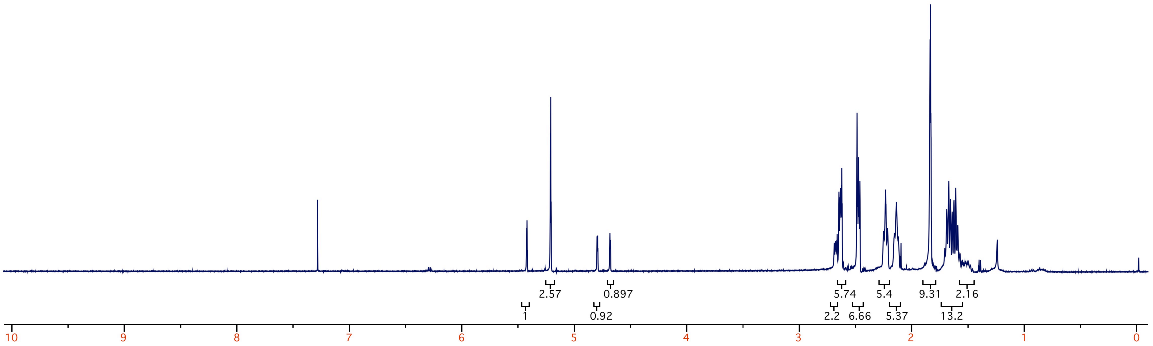

Supplement: File 1 — General methods, characterization data and NMR spectra of synthesized compounds. [file Beilstein_J_Org_Chem-09-2537-s001.pdf]
